# Supplementary material for: Allosteric regulation and crystallographic fragment screening of SARS-CoV-2 NSP15 endoribonuclease
Source: Nucleic Acids Res. 2023 Apr 28;51(10):5255–70. doi: 10.1093/nar/gkad314 (PMC10250223; doi:10.1093/nar/gkad314)
Supplement: gkad314_Supplemental_Files [file gkad314_supplemental_files.zip › Supplementary datasets.zip!/217/Refine_9/01-BUSTER/Cycle-4/LIST.html]

Refining

**BUSTER 2.X**: A suite of programs for macromolecular structure refinement by maximum-likelihood and Bayesian methods  
Copyright © 1992-2020 Global Phasing Ltd.  
All rights reserved.  
Authors: G. Bricogne, E. Blanc, M. Brandl, C. Flensburg, P. Keller, W. Paciorek, P. Roversi, O. Smart, C. Vonrhein, T. Womack  

Please reference use of the software as:

- BUSTER-TNT 2.X, Global Phasing Ltd, Sheraton House, Cambridge CB3 0AX, UK

Version: 2.10.3 for linux64-ifort made on May 20 2020 at 01:14:34  
Licenced to: Diamond Light Source, Scientific Software  

```
---


### Parsing of the BUSTER cardfile

TITLE : autoBUSTER refinement

   Cell information

   Symmetry information

 Using CCP4 X-ray form factors.

   Missing structure chemical composition

 Using ATOM block for chemical composition.


   Shell information

### Parsing of the TNT cardfile


---


### Reading observations file

 Number of reflexions in input:  47009

      Number of accepted reflexions:  42835

      Number of rejected reflexions:      0

   Reflexions in test set:            2171

   Reflexions in working set:        40664


---


### 1. Generating initial model

 2.10 * Shannon sampling rate. NX NY NZ:   288  288  192


  Effective Shannon rates are:    2.44    2.44    2.23


   Allocated BUFFER of size:     61.172 Mb.

   Initial Geometry Information


 No missing atoms declared


   Summary of model volumes

 No missing atoms declared: no prior computed


 Computing bulk solvent model scattering


 NCSMASK PDB radius for solvent mask:     2.15 Angstrom

 running PDBSET (setting cell and spacegroup P1) ... done
 running NCSMASK [1] (creating mask, SMOOTH 1.14715) ... done
 running MAPDUMP (to check if reduction to unit cell is needed) ... done
 running MAPMASK (converting mask to map) ... done

   Generation of babslv by theta-filter

   Electron density in frg mask:  0.420 Å-1

 FOBS  put on rough absolute scale by Wilson plot :
           K        =  0.71300E+02
           B        =     -5.561

 

### Cycle 0001: MaxLik scaling and refinement


   MaxLik Scaling Cycle 0001

   Wilson plot

   Statistics in resolution bins just after scaling
```

|  |  |
| --- | --- |
|  | - Rxpct factors plot- Rxpct factors table- <Log-Lik Gain>- Ln Sigma A- Correlation Coefficients |

```
   <Log-Likelihood> of null-hypothesis

Structure Factor Amplitudes


   Average Figure of Merit

 Switching off refinement of overall temperature factor


 Added B =     0.1632 Å2 to all atoms in fragment


 Added B =     0.1632 Å2 to B_SOLV

```
gelly refinement module
 Authors (2004-2009) O. S. Smart, C. Flensburg, W. Paciorek, C. Vonrhein, 
                     M. Brandl, T. O. Womack and G. Bricogne
         (2010-2011) O. S. Smart, C. Flensburg, W. Paciorek, C. Vonrhein, 
                     T. O. Womack and G. Bricogne
         (2012-2014) O. S. Smart, C. Flensburg, W. Paciorek, C. Vonrhein, 
                     and G. Bricogne
         (2015-2020) G. Bricogne, C. Flensburg, W. Paciorek, C. Vonrhein
 Copyright © 2004-2020 Global Phasing Ltd. 
 All rights reserved.
 gelly module compilation date May 20 2020 01:11:20
 
 gelly uses routines from TNT geometry. References: 
     Tronrud, D. E., Ten Eyck, L. F., Matthews, B. W. (1987)
     "An Efficient General-Purpose Least-Squares Refinement Program 
     for Macromolecular Structures". Acta Crystallogr A, 43:489-501.
     Tronrud, D. E. (1997). "The TNT Refinement Package", 
     in Macromolecular Crystallography, Part B, Eds Charlie Carter 
     and Robert Sweet, Methods in Enzymology, 277:306-319.
 wildcard matching function by Julian Robichaux http://www.nsftools.com

 No -random_seed flag found. Random number stream initialised using:
2147483562
2147483398

 Control flags set to:  -autoncs -hydrogens_input_occ2zero \
                     -hydrogens_output_occ2heavy -screen \
                     100 -screen_sigma \
                     3.0 -keeppdb \
                     1000000 -taomonitor_verbose \
                     2 
 

 ############### Starting setup via TNT routines

 Setting up tnt routines including license check:

                   TNT Stereochemistry Program
 Version: 5.22.3.0
 Licence for Diamond Light Source, Scientific Software is valid
 
 Using TNT geometry routines to load the tnt control file: gelly.tnt
 Using TNT routines to load initial coordinates from file fragment.cor
 Expecting 6517 atoms

 Found     2191 "NOTE BUSTER_RESIDUE_ATOM_DATA" cards
 Have copied information for    6517 atoms from TNT to gelly's store 
 
 The TNT WEIGHT cards used by this run:
 -----WEIGHT       BOND     2.0
 -----WEIGHT       ANGLE    2.0
 -----WEIGHT       IMPROPER 0.0
 -----WEIGHT       TORSION  2.0
 -----WEIGHT       PSEUDO   0.0
 -----WEIGHT       TRIGONAL 2.0
 -----WEIGHT       PLANE    5.0
 -----WEIGHT       CONTACT  5.0
 -----WEIGHT       BCORREL  10.0
 -----WEIGHT       CHIRAL   5.0
 -----WEIGHT       NCS      50.0
 -----WEIGHT       IDEAL    4.0
 -----WEIGHT       RFACTOR  9.88
 
 Dictionary versions picked up from NOTE BUSTER_RESTRAINT_DICT cards as: 
 ------ protgeo_eh99.dat (V1.6.4.17) 20191001  STANDARD AMINO ACID DICTIONARY. BONDS AND ANGLES FROM ENGH AND HUBER EH99. OTHER VALUES BASED ON PREVIOUS TNT OR TAKEN FROM CCP4. INCLUDES HYDROGEN ATOMS.
 ------ exoticaa.dat (V1.3.4.15) 20200327  COLLECTION OF NON-STANDARD AMINO ACIDS, MAINLY EH91 WITHOUT IDEAL DISTANCE INFO
 ------ nuclgeo.dat (V1.13.4.8) 20190909
 ------ bcorrel.dat (V1.15.10.2) 20190616
 ------ contact.dat (V1.15.12.10) 20191121
 ------ idealdist_contact.dat (V1.3.4.12) 20191001  IDEAL-DISTANCE CONTACT TERM DATA AS USED IN PROLSQ. VALUES USED HERE ARE BASED ON DATA HARVESTING OF PDB AS OF 20190109.
 ------ restraints for LIG (Unknown) from cif dictionary Z1269638430.cif
 ------ restraints for CIT (CITRIC ACID) from cif dictionary CIT.cif; generated by GRADE_PDB_LIGAND 1.2.12 (October 4 2016) using MOGUL 1.7.2(RC1), CSD as538be, with quantum mechanics RM1 ((DatabaseID PDB-CIT))
 ------ assume.dat (V1.9.4.2) 20180705
 
 Have picked up spacegroup name for pdb output as 'P 63      '
 Setting up Chain/Residue arrays
 
 Have command line specification -autoncs*
     Have found       1 NCS relations
     Pairwise matching residue number and type between chains: 
                    A        B        S        W    
          A         .      100%       -        -   
          B                  .        -        -   
          S                           .        -   
          W                                    .   
       Table key--                                                       
          all   : two chains have 100% match and same number of residues 
         multi  : ditto and they contribute to a multi chain ncs cluster 
          ***%  : two chains have >80% matching residue number/type      
           -    : chains appear to be unrelated                          
      chain A     have identified ncs to     1 other chains. So will weight each autoncs by (       6.666/#)=      6.6660
      chain B     have identified ncs to     1 other chains. So will weight each autoncs by (       6.666/#)=      6.6660
      chain S     have identified ncs to     0 other chains. 
      chain W     have identified ncs to     0 other chains. 
     For .Gelly cards produced see below - after pruning
 
 Setting up BUSTER_SET groups
 Have found    89 BUSTER_SET cards
 Now silently applying automatic/dictionary set definitions. For a verbose listing use '-verbose_set' flag.
 Applying user defined set definitions:
 =====NOTE BUSTER_SET ncsautoXcld = Water --> set 'ncsautoXcld' now has   966 atoms 
 =====NOTE BUSTER_SET ncsautoABset = Chain_A \ ncsautoXcld --> set 'ncsautoABset' now has  2781 atoms 
 Have defined    49 sets: 
      set:                 'All' has   6517 atoms in   1667 residues - automatically defined
      set:               'Empty' has      0 atoms in      0 residues - automatically defined
      set:            'ExoticAA' has      0 atoms in      0 residues - defined by dictionary file
      set:           'ExoticNUC' has      0 atoms in      0 residues - defined by dictionary file
      set:              'NotHET' has   5514 atoms in    698 residues - defined by dictionary file
      set:             'Chain_A' has   2781 atoms in    351 residues - automatically defined
      set:             'Chain_B' has   2770 atoms in    350 residues - automatically defined
      set:             'Chain_S' has    567 atoms in    567 residues - automatically defined
      set:             'Chain_W' has    399 atoms in    399 residues - automatically defined
      set:               'Water' has    966 atoms in    966 residues - defined by dictionary file
      set:          'StdProtein' has   5514 atoms in    698 residues - defined by dictionary file
      set:             'Protein' has   5514 atoms in    698 residues - defined by dictionary file
      set:                'Back' has   2794 atoms in    698 residues - defined by dictionary file
      set:                'Side' has   2720 atoms in    650 residues - defined by dictionary file
      set:        'RnaCanonical' has      0 atoms in      0 residues - defined by dictionary file
      set:        'DnaCanonical' has      0 atoms in      0 residues - defined by dictionary file
      set:     'RnaDnaCanonical' has      0 atoms in      0 residues - defined by dictionary file
      set:     'DnaRnaCanonical' has      0 atoms in      0 residues - defined by dictionary file
      set:         'RnaExtended' has      0 atoms in      0 residues - defined by dictionary file
      set:         'DnaExtended' has      0 atoms in      0 residues - defined by dictionary file
      set:      'RnaDnaExtended' has      0 atoms in      0 residues - defined by dictionary file
      set:      'DnaRnaExtended' has      0 atoms in      0 residues - defined by dictionary file
      set:                 'Dna' has      0 atoms in      0 residues - defined by dictionary file
      set:                 'Rna' has      0 atoms in      0 residues - defined by dictionary file
      set:              'RnaDna' has      0 atoms in      0 residues - defined by dictionary file
      set:              'DnaRna' has      0 atoms in      0 residues - defined by dictionary file
      set:             'Nucleic' has      0 atoms in      0 residues - defined by dictionary file
      set:             'Polymer' has   5514 atoms in    698 residues - defined by dictionary file
      set: 'BufferWeichenberger' has     26 atoms in      2 residues - defined by dictionary file
      set:    'BufferAdditional' has      0 atoms in      0 residues - defined by dictionary file
      set:              'Buffer' has     26 atoms in      2 residues - defined by dictionary file
      set:         'MetalAlkali' has      0 atoms in      0 residues - defined by dictionary file
      set:  'MetalAlkalineEarth' has      0 atoms in      0 residues - defined by dictionary file
      set:     'MetalTransition' has      0 atoms in      0 residues - defined by dictionary file
      set: 'MetalPostTransition' has      0 atoms in      0 residues - defined by dictionary file
      set:    'MetalLanthanides' has      0 atoms in      0 residues - defined by dictionary file
      set:               'Metal' has      0 atoms in      0 residues - defined by dictionary file
      set:         'SugarAlphaD' has      0 atoms in      0 residues - defined by dictionary file
      set:         'SugarAlphaL' has      0 atoms in      0 residues - defined by dictionary file
      set:               'Sugar' has      0 atoms in      0 residues - defined by dictionary file
      set:               'Other' has     37 atoms in      3 residues - defined by dictionary file
      set:            'Hydrogen' has      0 atoms in      0 residues - defined by dictionary file
      set:         'nonHydrogen' has   6517 atoms in   1667 residues - defined by dictionary file
      set:             'OccZero' has      0 atoms in      0 residues - defined by dictionary file
      set:          'OccNonZero' has   6517 atoms in   1667 residues - defined by dictionary file
      set:             'OccFull' has   6474 atoms in   1664 residues - defined by dictionary file
      set:          'OccNonFull' has     43 atoms in      3 residues - defined by dictionary file
      set:         'ncsautoXcld' has    966 atoms in    966 residues - user specified
      set:        'ncsautoABset' has   2781 atoms in    351 residues - user specified
 No command line arguments '-occ_allhet' or '-occ_restyp' so will not setup automated occcupancy refinement
 
 Interpreting CONSTANT and FREE cards: 
 =====CONSTANT     OCC
 Have found and interpreted      1 CONSTANT cards in the TNT input. 
 Before any COMBINE - Number of atoms with fixed XYZ =     0
 Before any COMBINE - Number of atoms with fixed B   =     0
 Before any COMBINE - Number of atoms with fixed OCC =  6517
 Before any COMBINE - Number of atoms with fixed BIJ =   966
                            cf total number of atoms =  6517
 
 There are no COMBINE cards to be found in the TNT card input 
 
 There are no NOTE BUSTER_COMBINE_RES cards to be found in the TNT card input 

 ############### Working out full connectivity list


 ############### Working out equivalent atoms


 Residue with most atoms given below:
        A|58:N(TRP)         
        A|58:CA(TRP)        
        A|58:CB(TRP)        
        A|58:CG(TRP)        
        A|58:CD1(TRP)       
        A|58:NE1(TRP)       
        A|58:CE2(TRP)       
        A|58:CD2(TRP)       
        A|58:CE3(TRP)       
        A|58:CZ3(TRP)       
        A|58:CH2(TRP)       
        A|58:CZ2(TRP)       
        A|58:C(TRP)         
        A|58:O(TRP)         


 Have no '-equiv_check_plus' argument - so will take atom type into
 account when checking for equivalence of otherwise identical atoms.
 

 ############### Looking at dihedrals


 ############### Looking at chirals

 NOTE BUSTER_CHIRAL_ANGLECUT card found - read value=    -31.00 degs from it
 NOTE BUSTER_CHIRAL_SIGMA card found - read value=      1.70 degs from it
 RANGE allowed for B variables in refinement:   from      3.00000 to    300.00000

 ############### Preparing for PDB output


 ############### Processing extra restraints explicitly defined

 No 'NOTE BUSTER_TARGET' cards or -target?? args found (used to read coordinates files for soft ncs to external)
 No 'NOTE BUSTER_OCCSUM'     cards found - so not using sum of occupancy restraints
 No 'NOTE BUSTER_DISTANCE'   cards found - so not setting up any additional atom-atom distance restraints
 No 'NOTE BUSTER_UTILANGLE'  cards found - so not setting up any additional restraints on any angle
 No 'NOTE BUSTER_UTILTOR'    cards found - so not setting up any additional sinusoidal restraints on torsions
 
 --- Have some similarity definitions 
     Similarity restraints on RMSD use superposition routines from: 
     Coutsias, E.A., Seok, C., Dill, K.A.(2004)
     "Using quaternions to calculate RMSD", J. Comput. Chem., 25:1849-1857 

 Have found    1 'NOTE BUSTER_SIM_DEFINE' cards 
     Maximum number of chains involved in any SIM group (excluding the template)=     1


 ########## Interpreting card: NOTE BUSTER_SIM_DEFINE ncsautoAB ncsautoABset B
     Number of atoms in template set=  2781
     Number of related chains (other than template) in SIM set=    1
     List of chains to be restrained (excluding template)=   B   
     Pairwise RMSD, XYZ in Angstroms, for SIM group: ncsautoAB
                 A         B   
     A         -----     ----- 
     B         0.635     ----- 
     Overall RMSD,XYZ for the SIM group is     0.635 Angstroms
     Pairwise rms deviation of [individual B's minus their set/chain average] in Angs**2
                 A         B         Bavg
     A         -----     -----    106.94
     B         50.79     -----     70.22
     Overall RMSD,B   for the SIM group is    50.792 Angs**2
     The number of common atoms for the above fits:
                 A         B   
     A         -----     ----- 
     B          2770     ----- 
     (compared to the template set that has    2781 atoms in it)
     List of atoms that are absent in one or more chains for this group: 
                                    A    B   
                    C01     501     +    -
                    C02     501     +    -
                    O03     501     +    -
                    N04     501     +    -
                    C05     501     +    -
                    C06     501     +    -
                    C07     501     +    -
                    N08     501     +    -
                    C09     501     +    -
                    C10     501     +    -
                    O11     501     +    -
     (key  +  atom present for this chain 
           -  atom absent  for this chain)
 transforms for each of the superpositions orthogonal matrix r11 r12 r13 r21 r22 r23 r31 r32 r33 tx ty tz (PDBSET syntax): 
TRANS_AB -0.872572 -0.487506 -0.030923 -0.487425  0.873100 -0.010638  0.032185  0.005790 -0.999465    33.902    33.109    14.620
 reverse transforms 
TRANS_BA -0.872572 -0.487425  0.032185 -0.487506  0.873100  0.005790 -0.030923 -0.010638 -0.999465    45.250   -12.464    16.012
 
 Setting up LSSR interatomic distance restraints
 Interpreting card: NOTE BUSTER_SIM_RESTRAIN_LSSR ncsautoAB 6.666
        setting up interatomic distance pair restraints with:
        Dmin=         0.00
        Dmax=         5.50
        sigma=        0.20
        Vmax=         3.00
        weight=       6.67
        number of distance pairs defined for this set is      35046
 Have not supplied either '-sim_swap_equiv' or '-sim_swap_equiv_plus' argument 
   so will not try swaping equivalent atoms to improve NCS and/or target agreement.
 
 NCS by LSSR outlier analysis
 -autoncs option automatic NCS prune routine -------- 
 Residues pruned out by gradient condition:
           A|23      A|27      A|30      A|34      A|54      A|87     A|135
          A|148     A|149     A|150     A|151     A|152     A|181     A|182
          A|183     A|184     A|185     A|186     A|187     A|189     A|216
          A|217     A|219     A|220     A|250     A|253     A|255     A|259
          A|264     A|310     A|319     A|328     A|344     A|345     A|401
           B|23      B|27      B|30      B|34      B|54      B|87     B|135
          B|148     B|151     B|152     B|181     B|182     B|183     B|184
          B|185     B|186     B|187     B|189     B|216     B|217     B|219
          B|220     B|250     B|253     B|255     B|259     B|264     B|310
          B|319     B|328     B|344     B|345     B|401
  
 Residues pruned out by function/max condition:
          A|149!    A|187!    A|188     A|220!    A|264!    A|346    A|ENDA 
          A|401!    B|149     B|187!    B|188     B|220!    B|264!    B|346 
         B|ENDA     B|401!
  
 Total number of residues for which LSSR restraints have been pruned out is       75 out of      700 participating
 Number of LSSR restraints pruned out is      5374 this is   15.3% of the total           35046
  
 -autoncs .Gelly cards suitable for adaptation for manual definition/pruning
           (note auto pruning is done in a slightly different way so exact results may differ slightly)
 -autoncs option .Gelly cards also written to file  auto_gelly_cards.txt
NOTE BUSTER_SET ncsautoXcld = Water    # card added - produced by -autoncs
NOTE BUSTER_SET ncsautoXcld = ncsautoXcld + { A|23 A|27 A|30 A|34 A|54 A|87 }    # card added - produced by -autoncs
NOTE BUSTER_SET ncsautoXcld = ncsautoXcld + { A|135 A|148 A|149 A|150 A|151 }    # card added - produced by -autoncs
NOTE BUSTER_SET ncsautoXcld = ncsautoXcld + { A|152 A|181 A|182 A|183 A|184 }    # card added - produced by -autoncs
NOTE BUSTER_SET ncsautoXcld = ncsautoXcld + { A|185 A|186 A|187 A|188 A|189 }    # card added - produced by -autoncs
NOTE BUSTER_SET ncsautoXcld = ncsautoXcld + { A|216 A|217 A|219 A|220 A|250 }    # card added - produced by -autoncs
NOTE BUSTER_SET ncsautoXcld = ncsautoXcld + { A|253 A|255 A|259 A|264 A|310 }    # card added - produced by -autoncs
NOTE BUSTER_SET ncsautoXcld = ncsautoXcld + { A|319 A|328 A|344 A|345 A|346 }    # card added - produced by -autoncs
NOTE BUSTER_SET ncsautoXcld = ncsautoXcld + { A|ENDA A|401 }    # card added - produced by -autoncs
NOTE BUSTER_SET ncsautoABset = Chain_A \ ncsautoXcld    # card added - produced by -autoncs
NOTE BUSTER_SIM_DEFINE ncsautoAB ncsautoABset B    # card added - produced by -autoncs
NOTE BUSTER_SIM_RESTRAIN_LSSR ncsautoAB 6.666    # card added - produced by -autoncs

 LSSR function contribution before pruning     45152.3
                             after pruning     10660.9
 Graph of which residues pruned from LSSR: ncs_pruned.mtv
 Graph of LSSR function outliers ncs_function_outliers.mtv
 Graph of LSSR gradient outliers ncs_gradient_outliers.mtv
 LSSR - analysis of distance difference distribution for NCS
         DeltaD less than 0.5 Angs:  proportion of population= 98.74% average DeltaD of subset     0.014 Angs
 Histogram of LSSR deltaD distribution ncs_histogram.mtv
 
 Interpreting card: NOTE BUSTER_TLS_SET tls1 { A|* }
 Interpreting card: NOTE BUSTER_TLS_SET tls2 { B|* }
 Interpreting card: NOTE BUSTER_TLS_SET tls1 { A|* }
 Interpreting card: NOTE BUSTER_TLS_T   tls1     -9.84122E-02  2.63375E-02  4.22310E-02 -5.87256E-02  1.84806E-02 -4.63797E-02
 Interpreting card: NOTE BUSTER_TLS_L   tls1      2.53184E+00  2.05865E+00  1.86779E+00 -8.79892E-01 -1.59062E+00  7.18718E-01
 Interpreting card: NOTE BUSTER_TLS_S   tls1     -1.17821E-02 -3.86196E-01  5.27491E-01 -4.35968E-01  2.67147E-01 -2.69276E-01  6.09961E-02 -3.35657E-01
 Interpreting card: NOTE BUSTER_TLS_O   tls1     -2.30832E+01 -8.41146E+01  1.12764E+01
 Interpreting card: NOTE BUSTER_TLS_SET tls2 { B|* }
 Interpreting card: NOTE BUSTER_TLS_T   tls2     -1.73365E-01 -1.10956E-01  1.17182E-01  1.49139E-02  2.71700E-02 -2.71783E-02
 Interpreting card: NOTE BUSTER_TLS_L   tls2      1.57079E+00  1.30451E+00  1.13623E+00  8.10314E-01 -1.11889E+00 -8.20951E-01
 Interpreting card: NOTE BUSTER_TLS_S   tls2     -7.19371E-02  1.20184E-01 -9.00838E-02  1.24667E-02  1.65761E-01  7.66243E-02 -1.29102E-01  7.22209E-02
 Interpreting card: NOTE BUSTER_TLS_O   tls2      2.22817E+01 -7.23258E+01  3.51644E+00
 Interpreting card: NOTE BUSTER_TLS_FIX tls1 ALL
 Interpreting card: NOTE BUSTER_TLS_FIX tls2 ALL


 TLS information. Total number of groups:     2


 Group   Natoms    FixRB   FixTLS   Group Tag    Group Spec
     1     2781      Y        Y     tls1    { A|* }
     2     2770      Y        Y     tls2    { B|* }
 
NOTE BUSTER_TLS_SET tls1 { A|* }
NOTE BUSTER_TLS_T   tls1     -9.84122E-02  2.63375E-02  4.22310E-02 -5.87256E-02  1.84806E-02 -4.63797E-02
NOTE BUSTER_TLS_L   tls1      2.53184E+00  2.05865E+00  1.86779E+00 -8.79892E-01 -1.59062E+00  7.18718E-01
NOTE BUSTER_TLS_S   tls1     -1.17821E-02 -3.86196E-01  5.27491E-01 -4.35968E-01  2.67147E-01 -2.69276E-01  6.09961E-02 -3.35657E-01
NOTE BUSTER_TLS_O   tls1     -2.30832E+01 -8.41146E+01  1.12764E+01
NOTE BUSTER_TLS_FIX tls1 ALL
 
NOTE BUSTER_TLS_SET tls2 { B|* }
NOTE BUSTER_TLS_T   tls2     -1.73365E-01 -1.10956E-01  1.17182E-01  1.49139E-02  2.71700E-02 -2.71783E-02
NOTE BUSTER_TLS_L   tls2      1.57079E+00  1.30451E+00  1.13623E+00  8.10314E-01 -1.11889E+00 -8.20951E-01
NOTE BUSTER_TLS_S   tls2     -7.19371E-02  1.20184E-01 -9.00838E-02  1.24667E-02  1.65761E-01  7.66243E-02 -1.29102E-01  7.22209E-02
NOTE BUSTER_TLS_O   tls2      2.22817E+01 -7.23258E+01  3.51644E+00
NOTE BUSTER_TLS_FIX tls2 ALL


 TLS: mmCIF group specification begin


#
loop_
_pdbx_refine_tls_group.id
_pdbx_refine_tls_group.refine_tls_id
_pdbx_refine_tls_group.beg_PDB_ins_code
_pdbx_refine_tls_group.beg_auth_asym_id
_pdbx_refine_tls_group.beg_auth_seq_id
_pdbx_refine_tls_group.end_PDB_ins_code
_pdbx_refine_tls_group.end_auth_asym_id
_pdbx_refine_tls_group.end_auth_seq_id
_pdbx_refine_tls_group.selection
_pdbx_refine_tls_group.selection_details
   1   1 . A   -1 . A  346 . '{ A|* }'
   2   1 . A  401 . A  501 . '{ A|* }'
   3   2 . B   -1 . B  346 . '{ B|* }'
   4   2 . B  401 . B  401 . '{ B|* }'
#


 TLS: mmCIF group specification end


 Interpreting card: NOTE BUSTER_ANISO_SET Empty
 Interpreting card: NOTE BUSTER_ANISO_WEIGHT SCA  16.0
 Interpreting card: NOTE BUSTER_ANISO_WEIGHT SPH   0.6    0.6
 Interpreting card: NOTE BUSTER_ANISO_WEIGHT SIM   7.8    4.8    2.6    0
 Interpreting card: NOTE BUSTER_ANISO_WEIGHT RIG  21.5   21.5    3.7    0


 After TLS and ANISO: Number of atoms with fixed/combined xyz =     0
 After TLS and ANISO: Number of atoms with fixed/combined B   =     0
 After TLS and ANISO: Number of atoms with fixed/combined OCC =  6517
 After TLS and ANISO: Number of atoms with fixed/combined BIJ =  6517
 After TLS and ANISO: Number of atoms in a TLS group          =  5551
                                     cf total number of atoms =  6517
 

 ############### Setup state vector

 No free_vect_ulim_xyz argument found: using default of  100.000
 No free_vect_llim_xyz argument found: using default of -100.000
 Number of parameters added for TLS description                 =       0
 Number of parameters to describe molecular conformation        =   26068

 ############### Setup free vector

 Number of free parameters to be adjusted in refinement   =   26068

 ############### Setting up own geometry


 No command line argument '-tnt_fn' so will use gelly to do geometry function calculations

 ============= Call TNT functions


 ============= Get TNT symmetry information

 
      Getting symmetry operators from TNT. 
          gelly will classify symmetry using pdb-like convention: 
               SYMOP   SYMMETRY
              NNNMMM   OPERATOR
                1555   X,Y,Z
                2555   -Y,X-Y,Z
                3555   -X+Y,-X,Z
                4555   -X,-Y,1/2+Z
                5555   Y,-X+Y,1/2+Z
                6555   X-Y,X,1/2+Z
          where NNN -> operator number and MMM -> translation vector
 

 ============= Own Gelly geometry/restraints setup


 ----------- Identify bond-types


    Setting up GEOMBONDREC_TYPE_HASH* to be able to map a given bond type (e.g.
    "single") to an internal integer (GBOND_TYPE=1) etc.

    Using default  GEOMBONDREC_TYPE_HASH_STR='SING* 1 DOUB* 2 TRIP* 3 AROM* 4 DELO* 4 METAL* 5':
    to override default use a NOTE BUSTER_GEOMBONDREC_TYPE_HASH_STR gelly card.

 ----------- Classify torsion into peptide omega and other


 ----------- Setup of non-bonded terms

 Ideal-distance contact term setup:
    Restraint function constants (normally these values are from NOTE BUSTER_IDEAL_CONTACT_* cards)
    Sigma for general contacts      0.200 Angs
    Sigma for 1-4 contacts          0.100 Angs (contacts across a torsion)
    Sigma for 1-5 contacts          0.150 Angs
    Sigma for 1-N contacts          0.200 Angs
    Sigma for HBOND pairs           0.100 Angs (contacts between HB_types A and D or B's)
    Contact distance increment for each atom in HBOND          -0.150 Angs 
    Contact distance increment for H-bond acceptor              0.100 Angs 
    Contact distance increment for 1-4 with an O atom          -0.050 Angs 
    Contact distance increment for 1-4 with an N atom          -0.180 Angs 
    Contact distance increment for 1-4 with an C atom          -0.370 Angs 
    Contact distance increment for 1-4 with an S atom          -0.150 Angs 
    Contact distance increment for 1-4 with an P atom          -0.150 Angs 
    Contact distance increment for 1-4 with any other atom     -0.150 Angs 
    All atoms matched for CCP4 atom type.
    Number of CCP4 atom types used is         24 (H-bound and not H-bound counted separately)

 Setting up short/ideal-distance contacts
   Using gelly special position handling. (For TNT instead rerun with argument '-special_tnt')

   No command line argument '-special_all' specified. We will auto-generate
   EXCLUDE cards only for atom-atom contacts across symmetry (i.e. on special
   position) when this involves a contact within a residue to itself.

   Have set up non-bonded exclusions - 1-3 contacts
   Atom A|27:CA         makes the most 1-3 contacts:  6

   Have set up non-bonded exclusions - 1-4 contacts
   Atom A|27:N          makes the most 1-4 contacts:  7

   Have set up non-bonded exclusions - 1-5 contacts
   Atom A|28:C.A        makes the most 1-5 contacts: 10

 ############################################################################################################
 ###################################################### setting up contact terms
 ############################################################################################################


 ============= Call Gelly versions of TNT functions

 WARNING: 1-4 contact without 1-(2,3)-4 data for A|401:C2(CIT)        and A|401:O6(CIT)        X-X
 WARNING: 1-4 contact without 1-(2,3)-4 data for A|401:O7(CIT)        and A|401:C5(CIT)        X-X
 WARNING: 1-4 contact without 1-(2,3)-4 data for A|401:O7(CIT)        and A|401:O5(CIT)        X-X
 WARNING: 1-4 contact without 1-(2,3)-4 data for A|401:C4(CIT)        and A|401:O6(CIT)        X-X
 WARNING: 1-4 contact without 1-(2,3)-4 data for A|501:C01(LIG)       and A|501:C05(LIG)       X-X
 WARNING: 1-4 contact without 1-(2,3)-4 data for A|501:C06(LIG)       and A|501:C09(LIG)       X-X
 WARNING: 1-4 contact without 1-(2,3)-4 data for A|501:C07(LIG)       and A|501:C10(LIG)       X-X
 WARNING: 1-4 contact without 1-(2,3)-4 data for B|401:C1(CIT)        and B|401:C4(CIT)        X-X
 WARNING: 1-4 contact without 1-(2,3)-4 data for B|401:C2(CIT)        and B|401:O6(CIT)        X-X
 WARNING: 1-4 contact without 1-(2,3)-4 data for B|401:O7(CIT)        and B|401:O5(CIT)        X-X
 WARNING: 1-4 contact without 1-(2,3)-4 data for B|401:C4(CIT)        and B|401:O6(CIT)        X-X

       TERM      weight      number      TNT_fn    Gelly_fn        diff
       BOND       2.000        5665    1811.273    1811.273       0.000
       ANGL       2.000        7687    5483.849    5483.849       0.000
       IMPR       0.000           0       0.000       0.000       0.000
       TORS       2.000        1973    3352.340    3352.340      -0.000
       TRIG       2.000           0       0.000       0.000       0.000
       PLAN       5.000         967    2551.117    2551.117       0.000
       CONT       5.000           0     176.560       0.000     176.560
       BCOR      10.000        5630    8062.996   13675.379   -5612.383


 ============= Call Gelly geometry functions

   Switching torsion functional form to sinusoidal rather than split harmonic functional.
      if you want this not to happen then rerun with '-torsharm'
      Weight for sinusoidal torsion=     2.000
   BCOR function/gradient will be split between H and non-H atoms (as for any atom pair). Use '-bcorrel_all_in_hydrogens' to change this.

 ----------- Fix B-correlation restraints

 
 Checking for bonds that do not have a bcorrel restraint defined
 We will wipe all existing BCORrels if OCC=0.0 atom(s) is/are involved. This can be' switched off via the '-bcorrel_nowipe_zeroocc' flag.
 We will wipe all existing BCORrels if only non-hydrogen atom(s) with fixed B(iso) are involved. This can be switched off via the '-bcorrel_nowipe_fixb_nonhydrogens' flag.
 We will keep all existing BCORrels if hydrogen atom(s) with fixed B(iso) is/are involved. This can be switched off via the '-bcorrel_wipe_fixb_hydrogens' flag.
 No -bcorrel_missing_sigma found. So will set sigma to     2.5 Ang**2 for any bond with no existing BCORrel
 No -bcorrel_missing_sigma_hydrogens found. So will set sigma to     1.0 Ang**2 for any bond with no existing BCORrel involving hydrogens

 No active BCORels found involving OCC=0.0 atom(s)


 No active BCORels found involving fixed-B (only) non-hydrogen atom(s)

    add a default BCOR for bond A|401:C1(CIT) A|401:C2(CIT)
    add a default BCOR for bond A|401:C1(CIT) A|401:O1(CIT)
    add a default BCOR for bond A|401:C1(CIT) A|401:O2(CIT)
    add a default BCOR for bond A|401:C2(CIT) A|401:C3(CIT)
    add a default BCOR for bond A|401:C3(CIT) A|401:C4(CIT)
    add a default BCOR for bond A|401:C3(CIT) A|401:C6(CIT)
    add a default BCOR for bond A|401:C3(CIT) A|401:O7(CIT)
    add a default BCOR for bond A|401:C4(CIT) A|401:C5(CIT)
    add a default BCOR for bond A|401:C5(CIT) A|401:O3(CIT)
    add a default BCOR for bond A|401:C5(CIT) A|401:O4(CIT)
    add a default BCOR for bond A|401:C6(CIT) A|401:O5(CIT)
    add a default BCOR for bond A|401:C6(CIT) A|401:O6(CIT)
    add a default BCOR for bond A|501:C01(LIG) A|501:C02(LIG)
    add a default BCOR for bond A|501:C02(LIG) A|501:O03(LIG)
    add a default BCOR for bond A|501:C02(LIG) A|501:N04(LIG)
    add a default BCOR for bond A|501:N04(LIG) A|501:C05(LIG)
    add a default BCOR for bond A|501:C05(LIG) A|501:C06(LIG)
    add a default BCOR for bond A|501:C06(LIG) A|501:C07(LIG)
    add a default BCOR for bond A|501:C06(LIG) A|501:O11(LIG)
    add a default BCOR for bond A|501:C07(LIG) A|501:N08(LIG)
    add a default BCOR for bond A|501:N08(LIG) A|501:C09(LIG)
    add a default BCOR for bond A|501:C09(LIG) A|501:C10(LIG)
    add a default BCOR for bond A|501:C10(LIG) A|501:O11(LIG)
    add a default BCOR for bond B|401:C1(CIT) B|401:C2(CIT)
    add a default BCOR for bond B|401:C1(CIT) B|401:O1(CIT)
    add a default BCOR for bond B|401:C1(CIT) B|401:O2(CIT)
    add a default BCOR for bond B|401:C2(CIT) B|401:C3(CIT)
    add a default BCOR for bond B|401:C3(CIT) B|401:C4(CIT)
    add a default BCOR for bond B|401:C3(CIT) B|401:C6(CIT)
    add a default BCOR for bond B|401:C3(CIT) B|401:O7(CIT)
    add a default BCOR for bond B|401:C4(CIT) B|401:C5(CIT)
    add a default BCOR for bond B|401:C5(CIT) B|401:O3(CIT)
    add a default BCOR for bond B|401:C5(CIT) B|401:O4(CIT)
    add a default BCOR for bond B|401:C6(CIT) B|401:O5(CIT)
    add a default BCOR for bond B|401:C6(CIT) B|401:O6(CIT)

 Have added a total of        35 BCORrels for bonds which do not already have a BCORrel


 No bonds (for potential BCORrel addition) were skipped due to both atoms having constant/fixed B(iso)


 ----------- Deactivate hydrogen-free planes if hydrogen atoms present

 A total of       0 planes have been deactivated because they are duplicated hydrogen-free and with-hydrogen forms.

 ----------- Handle peptide omega angle(s)

      Torsion angles split into peptide omega and other
         Number of peptide omega angles is       698
         Number of other torsion is             1275
      Peptide omega angles are monitored rather than directly restrained (PLANE used instead).

 ----------- Fix cis-PRO

 Now apply the fix to get correct restraints for cis PRO residues
   Have got      11 bonds records and      26 angles
   from GEOMETRY cards for the special PROC residue
   Weight for ideal-distance contact term=     4.000

 ----------- Prepare SSBOND/LINK for output PDB file


 ----------- Prepare for QM


 ----------- Prepare symmetry in utility distance restraints


 ############### Setting up Rhofit contacts (if needed)

 No ligand-(fixed protein) contact term will be used

 ############### Working out initial function and gradient


   MaxLik Scaling Cycle 0002
 
 Initial functional value = 4.926750E+05 rms gradient=      82.4792
 X-ray contribution to function value = 4.431060E+05
 Geometry contribution (inc ncs)      =      49568.9
 Geometry function summary
     term                           number     weight       rms        GooF      function     fn/numb
     ================================================================================================
     BOND bond lengths (angs)       5665       2.000      0.0080       0.400      1811.27       0.320
     ANGL bond angles (degs)        7687       2.000        1.00       0.597      5483.85       0.713
     TORS split-harmonic tors       1275       0.000       18.24       1.147         0.00       0.000
     SINTOR sinusoidal tors         1275       2.000       18.24                  2519.40       1.976
     PLAN general plane (angs)       967       5.000      0.0145       0.726      2551.12       2.638
     BCORrelations (angs^2)         5665      10.000       1.377       0.492     13685.18       2.416
     CONT (bad contacts)               0       5.000                                 0.00
     IDEAL(ideal-dist contact)      5717       4.000                             12807.48
     CHIRAL (gelly semiharmon)       740       5.000                                49.68
     SIM similarity restraint for NCS and/or TARGET                              10660.94
     OCCSUM restraint on sum of occupanies                                           0.00       as none defined
     DISTAN utility restraints interatomic distance                                  0.00       as none defined
     UTILANGLE utility angle restraints                                              0.00       as none defined
     UTILTOR utility restraints on torsion angles                                    0.00       as none defined
     Peptide omega torsion angles                           2.92 degs - monitor only

 Weighted rms      0.0080         1.002  (temporary rms bond and angle for BUSTER to pick up!)
 Weighted intra-residual rms(bond) for StdProtein (excluding H)      0.0078
 Weighted intra-residual rms(bond) for Buffer     (excluding H)      0.0032

 ############### Running initial check


 Initial Geometry Sanity Check

 Check for duplicate bonds: no duplicates found
 
 Check for duplicate or missing bond angles: all are OK
 Check for bond between an atom and itself not involving symmetry:  all are OK
   There are no bad contacts where Delta/sigma is greater than     6.5
   Worst contact dist=   2.944 std=   3.802 sigma=   0.206 abs(Delta/sigma)=    4.163 ats A|104:CE (MET) B|23:CB (PRO)
   It is likely that the geometry libraries, sequence info and links match the input molecule
 GEOMETRY CHECK OVERALL:  PASS


 ############### Creating geometry/screen report


 ############### Option "tao" - run Tao optimizer

 TAO: Toolkit for Advanced Optimization. Reference: 
     Munson, T., Sarich, J., Wild, S., Benson, S., McInnes, L.C. (2018)
     "TAO User Manual (Revision 3.9)", Mathematics and Computer Science
     Division, Argonne National Laboratory
     ANL/MCS-TM-322, http://www.mcs.anl.gov/petsc/
 
 Have found -keeppdb flag so will write out a .pdb file every 1000000 its during opt
 Will apply TAO optimization 
 Maximum number of iterations      100
 Convergence test on Grms       4.0000 (-glim)
 Limit on rms displacement to original position (rmsD2init): NONE test inactive (-dlim)

    analysing argument no 0 : -autoncs with j=0
    analysing argument no 1 : -hydrogens_input_occ2zero with j=0
    analysing argument no 2 : -hydrogens_output_occ2heavy with j=0
    analysing argument no 3 : -screen with j=0
    analysing argument no 4 : 100 with j=0
    analysing argument no 5 : -screen_sigma with j=0
    analysing argument no 6 : 3.0 with j=0
    analysing argument no 7 : -keeppdb with j=0
    analysing argument no 8 : 1000000 with j=0
    analysing argument no 9 : -taomonitor_verbose with j=0
    found argument starting with "-tao" : -taomonitor_verbose
    analysing argument no 10 : 2 with j=1
    found value after -tao/-options_/-viewer_ argument : "2"
 PETSC/TAO: setting option "-taomonitor_verbose" = "2"


   MaxLik Scaling Cycle 0003
   Iters     Tot_Funct    Xray_Funct    Geom_Funct          Grms     rmsD2init     maxD2init             R         Rfree           LLG       LLGfree     rms(bond)    rms(angle)       Opttag_H
   Iters             K   B_IMPF_FRAG        K_MISS   B_TEMP_MISS   K_IMPF_MISS   B_IMPF_MISS        K_SOLV   B_TEMP_SOLV   K_IMPF_SOLV   B_IMPF_SOLV        B_TEMP     B_TEMP_11     B_TEMP_12     B_TEMP_22     B_TEMP_13     B_TEMP_23     B_TEMP_33      K_WILSON      B_WILSON      FGCYCLES       Scatag_H
   Iters          BOND         ANGLE      IMPROPER       TORSION    SINTORSION      TRIGONAL        PLANAR  BCORRELATION       CONTACT     IDEALDIST        CHIRAL    SIMILARITY       OCCSSUM  UTILDISTANCE     UTILANGLE   UTILTORSION        RHOFIT        ADP-01        ADP-02        ADP-03        ADP-04        ADP-05        ADP-06        ADP-07        ADP-08        ADP-09        ADP-10      RHOFITPC            QM       Funtag_H
       0  4.925094E+05  4.431051E+05       49404.3       78.9091  0.000000E+00  0.000000E+00      0.186692      0.239908  0.000000E+00  0.000000E+00    0.00801671       1.00218       Opttag_D
       0       82.4536       1.25040       1.00000  0.000000E+00       1.00000  0.000000E+00      0.944096       93.7324       1.00000       39.5015  0.000000E+00       8.88686  0.000000E+00       8.88686  0.000000E+00  0.000000E+00      -17.7737       84.2583       3.97569        2       Scatag_D
       0       1811.27       5483.85  0.000000E+00  0.000000E+00       2519.40  0.000000E+00       2551.12       13520.6  0.000000E+00       12807.5       49.6774       10660.9 -1.000000E+10 -1.000000E+10 -1.000000E+10 -1.000000E+10 -1.000000E+10  0.000000E+00  0.000000E+00  0.000000E+00  0.000000E+00  0.000000E+00  0.000000E+00  0.000000E+00  0.000000E+00  0.000000E+00  0.000000E+00  0.000000E+00  0.000000E+00       Funtag_D
   Geometry Restraint Deviations SCREEN Information

 

   MaxLik Scaling Cycle 0004

   MaxLik Scaling Cycle 0005

   MaxLik Scaling Cycle 0006

   Wilson plot

   Statistics in resolution bins after refinement cycle 0001
```


|  |  |
| --- | --- |
|  | - Rxpct factors plot- Rxpct factors table- <Log-Lik Gain>- Ln Sigma A- Correlation Coefficients |


```
   Iters     Tot_Funct    Xray_Funct    Geom_Funct          Grms     rmsD2init     maxD2init             R         Rfree           LLG       LLGfree     rms(bond)    rms(angle)
   Iters             K   B_IMPF_FRAG        K_MISS   B_TEMP_MISS   K_IMPF_MISS   B_IMPF_MISS        K_SOLV   B_TEMP_SOLV   K_IMPF_SOLV   B_IMPF_SOLV        B_TEMP     B_TEMP_11     B_TEMP_12     B_TEMP_22     B_TEMP_13     B_TEMP_23     B_TEMP_33      K_WILSON      B_WILSON      FGCYCLES
   Iters          BOND         ANGLE      IMPROPER       TORSION    SINTORSION      TRIGONAL        PLANAR  BCORRELATION       CONTACT     IDEALDIST        CHIRAL    SIMILARITY       OCCSSUM  UTILDISTANCE     UTILANGLE   UTILTORSION        RHOFIT        ADP-01        ADP-02        ADP-03        ADP-04        ADP-05        ADP-06        ADP-07        ADP-08        ADP-09        ADP-10      RHOFITPC            QM
       1  4.912993E+05  4.430193E+05       48280.0       53.0150  3.055198E-04    0.00206908      0.186654      0.240160  3.297796E-04   -0.00104617    0.00803052       1.00236       Opttag_D
       1       82.4391       1.24656       1.00000  0.000000E+00       1.00000  0.000000E+00      0.944148       93.6380       1.00000       39.4813  0.000000E+00       8.89234  0.000000E+00       8.89234  0.000000E+00  0.000000E+00      -17.7847       84.2583       3.97569        3       Scatag_D
       1       1817.52       5485.78  0.000000E+00  0.000000E+00       2519.37  0.000000E+00       2550.86       12384.0  0.000000E+00       12810.2       49.7682       10662.6 -1.000000E+10 -1.000000E+10 -1.000000E+10 -1.000000E+10 -1.000000E+10  0.000000E+00  0.000000E+00  0.000000E+00  0.000000E+00  0.000000E+00  0.000000E+00  0.000000E+00  0.000000E+00  0.000000E+00  0.000000E+00  0.000000E+00  0.000000E+00       Funtag_D
   Geometry Restraint Deviations SCREEN Information

 

   MaxLik Scaling Cycle 0007

   Wilson plot

   Statistics in resolution bins after refinement cycle 0002
```


|  |  |
| --- | --- |
|  | - Rxpct factors plot- Rxpct factors table- <Log-Lik Gain>- Ln Sigma A- Correlation Coefficients |


```
   Iters     Tot_Funct    Xray_Funct    Geom_Funct          Grms     rmsD2init     maxD2init             R         Rfree           LLG       LLGfree     rms(bond)    rms(angle)
   Iters             K   B_IMPF_FRAG        K_MISS   B_TEMP_MISS   K_IMPF_MISS   B_IMPF_MISS        K_SOLV   B_TEMP_SOLV   K_IMPF_SOLV   B_IMPF_SOLV        B_TEMP     B_TEMP_11     B_TEMP_12     B_TEMP_22     B_TEMP_13     B_TEMP_23     B_TEMP_33      K_WILSON      B_WILSON      FGCYCLES
   Iters          BOND         ANGLE      IMPROPER       TORSION    SINTORSION      TRIGONAL        PLANAR  BCORRELATION       CONTACT     IDEALDIST        CHIRAL    SIMILARITY       OCCSSUM  UTILDISTANCE     UTILANGLE   UTILTORSION        RHOFIT        ADP-01        ADP-02        ADP-03        ADP-04        ADP-05        ADP-06        ADP-07        ADP-08        ADP-09        ADP-10      RHOFITPC            QM
       2  4.908825E+05  4.429137E+05       47968.8       68.7274  8.485763E-04    0.00667068      0.186614      0.240189  5.926042E-04   -0.00140108    0.00808642       1.00741       Opttag_D
       2       82.4117       1.24098       1.00000  0.000000E+00       1.00000  0.000000E+00      0.944164       93.5220       1.00000       39.4358  0.000000E+00       8.90145  0.000000E+00       8.90145  0.000000E+00  0.000000E+00      -17.8029       84.2583       3.97569        1       Scatag_D
       2       1842.91       5541.20  0.000000E+00  0.000000E+00       2519.54  0.000000E+00       2551.57       11975.9  0.000000E+00       12817.3       49.9103       10670.4 -1.000000E+10 -1.000000E+10 -1.000000E+10 -1.000000E+10 -1.000000E+10  0.000000E+00  0.000000E+00  0.000000E+00  0.000000E+00  0.000000E+00  0.000000E+00  0.000000E+00  0.000000E+00  0.000000E+00  0.000000E+00  0.000000E+00  0.000000E+00       Funtag_D
   Geometry Restraint Deviations SCREEN Information

 
       3  4.905269E+05  4.428784E+05       47648.5       42.3744  9.804617E-04    0.00831733      0.186600      0.240189  6.805966E-04   -0.00147182    0.00808483       1.00425       Opttag_D
       3       82.4024       1.23918       1.00000  0.000000E+00       1.00000  0.000000E+00      0.944159       93.4883       1.00000       39.4229  0.000000E+00       8.90461  0.000000E+00       8.90461  0.000000E+00  0.000000E+00      -17.8092       84.2583       3.97569        1       Scatag_D
       3       1842.19       5506.43  0.000000E+00  0.000000E+00       2519.47  0.000000E+00       2552.33       11685.4  0.000000E+00       12820.1       50.0479       10672.6 -1.000000E+10 -1.000000E+10 -1.000000E+10 -1.000000E+10 -1.000000E+10  0.000000E+00  0.000000E+00  0.000000E+00  0.000000E+00  0.000000E+00  0.000000E+00  0.000000E+00  0.000000E+00  0.000000E+00  0.000000E+00  0.000000E+00  0.000000E+00       Funtag_D
       4  4.903498E+05  4.428383E+05       47511.5       32.3888    0.00116346     0.0102931      0.186587      0.240196  7.802125E-04   -0.00156515    0.00809466       1.00359       Opttag_D
       4       82.3912       1.23708       1.00000  0.000000E+00       1.00000  0.000000E+00      0.944153       93.4482       1.00000       39.4076  0.000000E+00       8.90849  0.000000E+00       8.90849  0.000000E+00  0.000000E+00      -17.8170       84.2583       3.97569        1       Scatag_D
       4       1846.67       5499.24  0.000000E+00  0.000000E+00       2519.52  0.000000E+00       2553.35       11543.4  0.000000E+00       12823.2       50.1712       10675.9 -1.000000E+10 -1.000000E+10 -1.000000E+10 -1.000000E+10 -1.000000E+10  0.000000E+00  0.000000E+00  0.000000E+00  0.000000E+00  0.000000E+00  0.000000E+00  0.000000E+00  0.000000E+00  0.000000E+00  0.000000E+00  0.000000E+00  0.000000E+00       Funtag_D
       5  4.901469E+05  4.427576E+05       47389.4       31.5005    0.00160225     0.0147742      0.186562      0.240216  9.811735E-04   -0.00180896    0.00812188       1.00422       Opttag_D
       5       82.3661       1.23260       1.00000  0.000000E+00       1.00000  0.000000E+00      0.944137       93.3603       1.00000       39.3723  0.000000E+00       8.91712  0.000000E+00       8.91712  0.000000E+00  0.000000E+00      -17.8342       84.2583       3.97569        1       Scatag_D
       5       1859.11       5506.11  0.000000E+00  0.000000E+00       2519.71  0.000000E+00       2556.04       11382.7  0.000000E+00       12830.9       50.3370       10684.5 -1.000000E+10 -1.000000E+10 -1.000000E+10 -1.000000E+10 -1.000000E+10  0.000000E+00  0.000000E+00  0.000000E+00  0.000000E+00  0.000000E+00  0.000000E+00  0.000000E+00  0.000000E+00  0.000000E+00  0.000000E+00  0.000000E+00  0.000000E+00       Funtag_D
       6  4.900232E+05  4.426363E+05       47386.8       57.4359    0.00246133     0.0231707      0.186530      0.240268    0.00128290   -0.00231377    0.00819932       1.01447       Opttag_D
       6       82.3192       1.22519       1.00000  0.000000E+00       1.00000  0.000000E+00      0.944083       93.2134       1.00000       39.3078  0.000000E+00       8.93235  0.000000E+00       8.93235  0.000000E+00  0.000000E+00      -17.8647       84.2583       3.97569        1       Scatag_D
       6       1894.73       5619.09  0.000000E+00  0.000000E+00       2519.90  0.000000E+00       2562.75       11190.7  0.000000E+00       12846.2       50.4599       10703.0 -1.000000E+10 -1.000000E+10 -1.000000E+10 -1.000000E+10 -1.000000E+10  0.000000E+00  0.000000E+00  0.000000E+00  0.000000E+00  0.000000E+00  0.000000E+00  0.000000E+00  0.000000E+00  0.000000E+00  0.000000E+00  0.000000E+00  0.000000E+00       Funtag_D
       7  4.898278E+05  4.426166E+05       47211.2       28.4971    0.00261568     0.0250184      0.186528      0.240285    0.00133218   -0.00244399    0.00817420       1.00550       Opttag_D
       7       82.3084       1.22376       1.00000  0.000000E+00       1.00000  0.000000E+00      0.944063       93.1854       1.00000       39.2937  0.000000E+00       8.93557  0.000000E+00       8.93557  0.000000E+00  0.000000E+00      -17.8711       84.2583       3.97569        1       Scatag_D
       7       1883.14       5520.25  0.000000E+00  0.000000E+00       2520.08  0.000000E+00       2564.67       11118.7  0.000000E+00       12849.2       50.4310       10704.7 -1.000000E+10 -1.000000E+10 -1.000000E+10 -1.000000E+10 -1.000000E+10  0.000000E+00  0.000000E+00  0.000000E+00  0.000000E+00  0.000000E+00  0.000000E+00  0.000000E+00  0.000000E+00  0.000000E+00  0.000000E+00  0.000000E+00  0.000000E+00       Funtag_D
       8  4.897358E+05  4.426035E+05       47132.3       24.9114    0.00278923     0.0267188      0.186528      0.240302    0.00136475   -0.00258400    0.00817225       1.00572       Opttag_D
       8       82.2978       1.22257       1.00000  0.000000E+00       1.00000  0.000000E+00      0.944037       93.1629       1.00000       39.2806  0.000000E+00       8.93837  0.000000E+00       8.93837  0.000000E+00  0.000000E+00      -17.8767       84.2583       3.97569        1       Scatag_D
       8       1882.24       5522.64  0.000000E+00  0.000000E+00       2520.20  0.000000E+00       2566.81       11032.1  0.000000E+00       12852.2       50.3591       10705.7 -1.000000E+10 -1.000000E+10 -1.000000E+10 -1.000000E+10 -1.000000E+10  0.000000E+00  0.000000E+00  0.000000E+00  0.000000E+00  0.000000E+00  0.000000E+00  0.000000E+00  0.000000E+00  0.000000E+00  0.000000E+00  0.000000E+00  0.000000E+00       Funtag_D
       9  4.896231E+05  4.425723E+05       47050.8       26.7844    0.00320642     0.0305939      0.186529      0.240340    0.00144246   -0.00287683    0.00817986       1.00669       Opttag_D
       9       82.2738       1.21982       1.00000  0.000000E+00       1.00000  0.000000E+00      0.943987       93.1056       1.00000       39.2517  0.000000E+00       8.94478  0.000000E+00       8.94478  0.000000E+00  0.000000E+00      -17.8896       84.2583       3.97569        1       Scatag_D
       9       1885.75       5533.27  0.000000E+00  0.000000E+00       2520.38  0.000000E+00       2571.58       10921.1  0.000000E+00       12859.0       50.2923       10709.5 -1.000000E+10 -1.000000E+10 -1.000000E+10 -1.000000E+10 -1.000000E+10  0.000000E+00  0.000000E+00  0.000000E+00  0.000000E+00  0.000000E+00  0.000000E+00  0.000000E+00  0.000000E+00  0.000000E+00  0.000000E+00  0.000000E+00  0.000000E+00       Funtag_D
      10  4.895164E+05  4.425276E+05       46988.7       42.3188    0.00403997     0.0377010      0.186535      0.240415    0.00155351   -0.00339728    0.00819718       1.01251       Opttag_D
      10       82.2288       1.21500       1.00000  0.000000E+00       1.00000  0.000000E+00      0.943896       92.9922       1.00000       39.2013  0.000000E+00       8.95639  0.000000E+00       8.95639  0.000000E+00  0.000000E+00      -17.9128       84.2583       3.97569        1       Scatag_D
      10       1893.74       5597.46  0.000000E+00  0.000000E+00       2520.70  0.000000E+00       2580.97       10758.4  0.000000E+00       12871.2       50.2795       10716.0 -1.000000E+10 -1.000000E+10 -1.000000E+10 -1.000000E+10 -1.000000E+10  0.000000E+00  0.000000E+00  0.000000E+00  0.000000E+00  0.000000E+00  0.000000E+00  0.000000E+00  0.000000E+00  0.000000E+00  0.000000E+00  0.000000E+00  0.000000E+00       Funtag_D
      11  4.893896E+05  4.425031E+05       46886.5       23.0784    0.00459300     0.0424192      0.186542      0.240462    0.00161470   -0.00372745    0.00819932       1.00772       Opttag_D
      11       82.1999       1.21198       1.00000  0.000000E+00       1.00000  0.000000E+00      0.943844       92.9143       1.00000       39.1691  0.000000E+00       8.96400  0.000000E+00       8.96400  0.000000E+00  0.000000E+00      -17.9280       84.2583       3.97569        1       Scatag_D
      11       1894.73       5544.58  0.000000E+00  0.000000E+00       2520.85  0.000000E+00       2587.12       10688.9  0.000000E+00       12879.1       50.3284       10720.9 -1.000000E+10 -1.000000E+10 -1.000000E+10 -1.000000E+10 -1.000000E+10  0.000000E+00  0.000000E+00  0.000000E+00  0.000000E+00  0.000000E+00  0.000000E+00  0.000000E+00  0.000000E+00  0.000000E+00  0.000000E+00  0.000000E+00  0.000000E+00       Funtag_D
      12  4.893223E+05  4.424952E+05       46827.1       19.4182    0.00482339     0.0441908      0.186545      0.240481    0.00163420   -0.00384361    0.00820373       1.00772       Opttag_D
      12       82.1882       1.21077       1.00000  0.000000E+00       1.00000  0.000000E+00      0.943828       92.8787       1.00000       39.1569  0.000000E+00       8.96698  0.000000E+00       8.96698  0.000000E+00  0.000000E+00      -17.9340       84.2583       3.97569        1       Scatag_D
      12       1896.77       5544.62  0.000000E+00  0.000000E+00       2520.87  0.000000E+00       2589.33       10622.2  0.000000E+00       12881.4       50.3988       10721.5 -1.000000E+10 -1.000000E+10 -1.000000E+10 -1.000000E+10 -1.000000E+10  0.000000E+00  0.000000E+00  0.000000E+00  0.000000E+00  0.000000E+00  0.000000E+00  0.000000E+00  0.000000E+00  0.000000E+00  0.000000E+00  0.000000E+00  0.000000E+00       Funtag_D
      13  4.892338E+05  4.424802E+05       46753.6       21.7136    0.00539159     0.0484448      0.186556      0.240525    0.00167157   -0.00410957    0.00821374       1.00873       Opttag_D
      13       82.1606       1.20798       1.00000  0.000000E+00       1.00000  0.000000E+00      0.943788       92.7937       1.00000       39.1286  0.000000E+00       8.97393  0.000000E+00       8.97393  0.000000E+00  0.000000E+00      -17.9479       84.2583       3.97569        1       Scatag_D
      13       1901.40       5555.72  0.000000E+00  0.000000E+00       2521.01  0.000000E+00       2594.50       10520.7  0.000000E+00       12886.7       50.5641       10723.0 -1.000000E+10 -1.000000E+10 -1.000000E+10 -1.000000E+10 -1.000000E+10  0.000000E+00  0.000000E+00  0.000000E+00  0.000000E+00  0.000000E+00  0.000000E+00  0.000000E+00  0.000000E+00  0.000000E+00  0.000000E+00  0.000000E+00  0.000000E+00       Funtag_D
      14  4.891964E+05  4.424628E+05       46733.5       37.0262    0.00630351     0.0556572      0.186575      0.240581    0.00171480   -0.00450766    0.00823971       1.01306       Opttag_D
      14       82.1195       1.20387       1.00000  0.000000E+00       1.00000  0.000000E+00      0.943724       92.6667       1.00000       39.0866  0.000000E+00       8.98442  0.000000E+00       8.98442  0.000000E+00  0.000000E+00      -17.9688       84.2583       3.97569        1       Scatag_D
      14       1913.45       5603.49  0.000000E+00  0.000000E+00       2521.31  0.000000E+00       2602.44       10418.9  0.000000E+00       12896.6       50.7475       10726.5 -1.000000E+10 -1.000000E+10 -1.000000E+10 -1.000000E+10 -1.000000E+10  0.000000E+00  0.000000E+00  0.000000E+00  0.000000E+00  0.000000E+00  0.000000E+00  0.000000E+00  0.000000E+00  0.000000E+00  0.000000E+00  0.000000E+00  0.000000E+00       Funtag_D
      15  4.891210E+05  4.424597E+05       46661.3       18.3529    0.00640966     0.0571299      0.186577      0.240584    0.00172258   -0.00453049    0.00822666       1.00938       Opttag_D
      15       82.1149       1.20336       1.00000  0.000000E+00       1.00000  0.000000E+00      0.943717       92.6513       1.00000       39.0817  0.000000E+00       8.98544  0.000000E+00       8.98544  0.000000E+00  0.000000E+00      -17.9709       84.2583       3.97569        1       Scatag_D
      15       1907.39       5562.84  0.000000E+00  0.000000E+00       2521.37  0.000000E+00       2602.95       10393.7  0.000000E+00       12896.6       50.7588       10725.7 -1.000000E+10 -1.000000E+10 -1.000000E+10 -1.000000E+10 -1.000000E+10  0.000000E+00  0.000000E+00  0.000000E+00  0.000000E+00  0.000000E+00  0.000000E+00  0.000000E+00  0.000000E+00  0.000000E+00  0.000000E+00  0.000000E+00  0.000000E+00       Funtag_D
      16  4.890739E+05  4.424519E+05       46622.0       15.3359    0.00669717     0.0606505      0.186581      0.240592    0.00174198   -0.00461427    0.00822751       1.00958       Opttag_D
      16       82.1031       1.20207       1.00000  0.000000E+00       1.00000  0.000000E+00      0.943696       92.6138       1.00000       39.0693  0.000000E+00       8.98829  0.000000E+00       8.98829  0.000000E+00  0.000000E+00      -17.9766       84.2583       3.97569        1       Scatag_D
      16       1907.78       5565.12  0.000000E+00  0.000000E+00       2521.53  0.000000E+00       2604.73       10347.3  0.000000E+00       12898.7       50.7709       10726.0 -1.000000E+10 -1.000000E+10 -1.000000E+10 -1.000000E+10 -1.000000E+10  0.000000E+00  0.000000E+00  0.000000E+00  0.000000E+00  0.000000E+00  0.000000E+00  0.000000E+00  0.000000E+00  0.000000E+00  0.000000E+00  0.000000E+00  0.000000E+00       Funtag_D
      17  4.890226E+05  4.424387E+05       46583.9       17.6728    0.00726655     0.0674721      0.186591      0.240607    0.00177483   -0.00478273    0.00823445       1.01043       Opttag_D
      17       82.0807       1.19965       1.00000  0.000000E+00       1.00000  0.000000E+00      0.943655       92.5418       1.00000       39.0463  0.000000E+00       8.99373  0.000000E+00       8.99373  0.000000E+00  0.000000E+00      -17.9875       84.2583       3.97569        1       Scatag_D
      17       1911.00       5574.50  0.000000E+00  0.000000E+00       2521.81  0.000000E+00       2608.34       10286.7  0.000000E+00       12903.6       50.8022       10727.1 -1.000000E+10 -1.000000E+10 -1.000000E+10 -1.000000E+10 -1.000000E+10  0.000000E+00  0.000000E+00  0.000000E+00  0.000000E+00  0.000000E+00  0.000000E+00  0.000000E+00  0.000000E+00  0.000000E+00  0.000000E+00  0.000000E+00  0.000000E+00       Funtag_D
      18  4.889500E+05  4.424141E+05       46535.9       24.2811    0.00831683     0.0802948      0.186603      0.240627    0.00183622   -0.00504576    0.00824566       1.01252       Opttag_D
      18       82.0435       1.19551       1.00000  0.000000E+00       1.00000  0.000000E+00      0.943583       92.4176       1.00000       39.0082  0.000000E+00       9.00239  0.000000E+00       9.00239  0.000000E+00  0.000000E+00      -18.0048       84.2583       3.97569        1       Scatag_D
      18       1916.21       5597.56  0.000000E+00  0.000000E+00       2522.35  0.000000E+00       2613.79       10193.9  0.000000E+00       12912.5       50.8389       10728.7 -1.000000E+10 -1.000000E+10 -1.000000E+10 -1.000000E+10 -1.000000E+10  0.000000E+00  0.000000E+00  0.000000E+00  0.000000E+00  0.000000E+00  0.000000E+00  0.000000E+00  0.000000E+00  0.000000E+00  0.000000E+00  0.000000E+00  0.000000E+00       Funtag_D
      19  4.888909E+05  4.423917E+05       46499.1       23.3481    0.00980199     0.0980578      0.186628      0.240660    0.00189174   -0.00541214    0.00826545       1.01302       Opttag_D
      19       81.9957       1.19031       1.00000  0.000000E+00       1.00000  0.000000E+00      0.943485       92.2518       1.00000       38.9609  0.000000E+00       9.01343  0.000000E+00       9.01343  0.000000E+00  0.000000E+00      -18.0269       84.2583       3.97569        1       Scatag_D
      19       1925.42       5603.13  0.000000E+00  0.000000E+00       2523.24  0.000000E+00       2621.13       10117.1  0.000000E+00       12926.0       50.9220       10732.2 -1.000000E+10 -1.000000E+10 -1.000000E+10 -1.000000E+10 -1.000000E+10  0.000000E+00  0.000000E+00  0.000000E+00  0.000000E+00  0.000000E+00  0.000000E+00  0.000000E+00  0.000000E+00  0.000000E+00  0.000000E+00  0.000000E+00  0.000000E+00       Funtag_D
      20  4.888407E+05  4.423810E+05       46459.6       13.5705    0.00990350     0.0997465      0.186623      0.240657    0.00191845   -0.00540899    0.00825934       1.01202       Opttag_D
      20       81.9951       1.18993       1.00000  0.000000E+00       1.00000  0.000000E+00      0.943481       92.2445       1.00000       38.9579  0.000000E+00       9.01327  0.000000E+00       9.01327  0.000000E+00  0.000000E+00      -18.0265       84.2583       3.97569        1       Scatag_D
      20       1922.57       5592.04  0.000000E+00  0.000000E+00       2523.31  0.000000E+00       2620.33       10092.7  0.000000E+00       12926.7       50.9374       10731.1 -1.000000E+10 -1.000000E+10 -1.000000E+10 -1.000000E+10 -1.000000E+10  0.000000E+00  0.000000E+00  0.000000E+00  0.000000E+00  0.000000E+00  0.000000E+00  0.000000E+00  0.000000E+00  0.000000E+00  0.000000E+00  0.000000E+00  0.000000E+00       Funtag_D
      21  4.887956E+05  4.423594E+05       46436.2       13.7237     0.0101971      0.104114      0.186615      0.240657    0.00197224   -0.00542743    0.00825913       1.01236       Opttag_D
      21       81.9918       1.18891       1.00000  0.000000E+00       1.00000  0.000000E+00      0.943470       92.2208       1.00000       38.9494  0.000000E+00       9.01349  0.000000E+00       9.01349  0.000000E+00  0.000000E+00      -18.0270       84.2583       3.97569        1       Scatag_D
      21       1922.47       5595.77  0.000000E+00  0.000000E+00       2523.55  0.000000E+00       2619.20       10065.7  0.000000E+00       12928.3       50.9838       10730.2 -1.000000E+10 -1.000000E+10 -1.000000E+10 -1.000000E+10 -1.000000E+10  0.000000E+00  0.000000E+00  0.000000E+00  0.000000E+00  0.000000E+00  0.000000E+00  0.000000E+00  0.000000E+00  0.000000E+00  0.000000E+00  0.000000E+00  0.000000E+00       Funtag_D
      22  4.887605E+05  4.423310E+05       46429.5       17.0534     0.0108554      0.112833      0.186614      0.240671    0.00204304   -0.00552313    0.00826137       1.01315       Opttag_D
      22       81.9794       1.18678       1.00000  0.000000E+00       1.00000  0.000000E+00      0.943438       92.1605       1.00000       38.9305  0.000000E+00       9.01571  0.000000E+00       9.01571  0.000000E+00  0.000000E+00      -18.0314       84.2583       3.97569        1       Scatag_D
      22       1923.52       5604.51  0.000000E+00  0.000000E+00       2524.06  0.000000E+00       2619.47       10043.4  0.000000E+00       12933.1       51.0625       10730.3 -1.000000E+10 -1.000000E+10 -1.000000E+10 -1.000000E+10 -1.000000E+10  0.000000E+00  0.000000E+00  0.000000E+00  0.000000E+00  0.000000E+00  0.000000E+00  0.000000E+00  0.000000E+00  0.000000E+00  0.000000E+00  0.000000E+00  0.000000E+00       Funtag_D
      23  4.887137E+05  4.422941E+05       46419.6       14.5478     0.0117870      0.124950      0.186612      0.240692    0.00213487   -0.00566573    0.00826439       1.01335       Opttag_D
      23       81.9634       1.18393       1.00000  0.000000E+00       1.00000  0.000000E+00      0.943398       92.0780       1.00000       38.9052  0.000000E+00       9.01872  0.000000E+00       9.01872  0.000000E+00  0.000000E+00      -18.0374       84.2583       3.97569        1       Scatag_D
      23       1924.92       5606.71  0.000000E+00  0.000000E+00       2524.73  0.000000E+00       2620.24       10020.2  0.000000E+00       12940.0       51.1562       10731.6 -1.000000E+10 -1.000000E+10 -1.000000E+10 -1.000000E+10 -1.000000E+10  0.000000E+00  0.000000E+00  0.000000E+00  0.000000E+00  0.000000E+00  0.000000E+00  0.000000E+00  0.000000E+00  0.000000E+00  0.000000E+00  0.000000E+00  0.000000E+00       Funtag_D
      24  4.886678E+05  4.422504E+05       46417.4       16.0010     0.0128478      0.138744      0.186607      0.240719    0.00224371   -0.00583407    0.00827138       1.01421       Opttag_D
      24       81.9491       1.18090       1.00000  0.000000E+00       1.00000  0.000000E+00      0.943354       91.9926       1.00000       38.8781  0.000000E+00       9.02130  0.000000E+00       9.02130  0.000000E+00  0.000000E+00      -18.0426       84.2583       3.97569        1       Scatag_D
      24       1928.18       5616.21  0.000000E+00  0.000000E+00       2525.51  0.000000E+00       2621.25       9992.96  0.000000E+00       12948.1       51.2100       10734.0 -1.000000E+10 -1.000000E+10 -1.000000E+10 -1.000000E+10 -1.000000E+10  0.000000E+00  0.000000E+00  0.000000E+00  0.000000E+00  0.000000E+00  0.000000E+00  0.000000E+00  0.000000E+00  0.000000E+00  0.000000E+00  0.000000E+00  0.000000E+00       Funtag_D
      25  4.886273E+05  4.422092E+05       46418.1       13.9976     0.0138150      0.151338      0.186601      0.240740    0.00234602   -0.00598476    0.00826971       1.01488       Opttag_D
      25       81.9397       1.17833       1.00000  0.000000E+00       1.00000  0.000000E+00      0.943316       91.9226       1.00000       38.8564  0.000000E+00       9.02303  0.000000E+00       9.02303  0.000000E+00  0.000000E+00      -18.0461       84.2583       3.97569        1       Scatag_D
      25       1927.40       5623.70  0.000000E+00  0.000000E+00       2526.18  0.000000E+00       2622.41       9975.68  0.000000E+00       12955.4       51.2042       10736.1 -1.000000E+10 -1.000000E+10 -1.000000E+10 -1.000000E+10 -1.000000E+10  0.000000E+00  0.000000E+00  0.000000E+00  0.000000E+00  0.000000E+00  0.000000E+00  0.000000E+00  0.000000E+00  0.000000E+00  0.000000E+00  0.000000E+00  0.000000E+00       Funtag_D
      26  4.885983E+05  4.421857E+05       46412.6       12.7454     0.0141971      0.156632      0.186594      0.240743    0.00240452   -0.00604380    0.00826884       1.01503       Opttag_D
      26       81.9407       1.17747       1.00000  0.000000E+00       1.00000  0.000000E+00      0.943303       91.9065       1.00000       38.8494  0.000000E+00       9.02267  0.000000E+00       9.02267  0.000000E+00  0.000000E+00      -18.0453       84.2583       3.97569        1       Scatag_D
      26       1926.99       5625.36  0.000000E+00  0.000000E+00       2526.45  0.000000E+00       2622.81       9964.38  0.000000E+00       12958.5       51.1567       10737.0 -1.000000E+10 -1.000000E+10 -1.000000E+10 -1.000000E+10 -1.000000E+10  0.000000E+00  0.000000E+00  0.000000E+00  0.000000E+00  0.000000E+00  0.000000E+00  0.000000E+00  0.000000E+00  0.000000E+00  0.000000E+00  0.000000E+00  0.000000E+00       Funtag_D
      27  4.885623E+05  4.421500E+05       46412.4       11.5287     0.0147442      0.164313      0.186579      0.240747    0.00249357   -0.00612488    0.00826863       1.01517       Opttag_D
      27       81.9451       1.17642       1.00000  0.000000E+00       1.00000  0.000000E+00      0.943283       91.8919       1.00000       38.8414  0.000000E+00       9.02154  0.000000E+00       9.02154  0.000000E+00  0.000000E+00      -18.0431       84.2583       3.97569        1       Scatag_D
      27       1926.90       5626.92  0.000000E+00  0.000000E+00       2526.77  0.000000E+00       2623.49       9955.81  0.000000E+00       12962.7       51.0899       10738.7 -1.000000E+10 -1.000000E+10 -1.000000E+10 -1.000000E+10 -1.000000E+10  0.000000E+00  0.000000E+00  0.000000E+00  0.000000E+00  0.000000E+00  0.000000E+00  0.000000E+00  0.000000E+00  0.000000E+00  0.000000E+00  0.000000E+00  0.000000E+00       Funtag_D
      28  4.885293E+05  4.421028E+05       46426.5       16.1349     0.0156288      0.176285      0.186561      0.240754    0.00261095   -0.00623914    0.00827149       1.01629       Opttag_D
      28       81.9503       1.17491       1.00000  0.000000E+00       1.00000  0.000000E+00      0.943251       91.8653       1.00000       38.8299  0.000000E+00       9.02023  0.000000E+00       9.02023  0.000000E+00  0.000000E+00      -18.0405       84.2583       3.97569        1       Scatag_D
      28       1928.23       5639.27  0.000000E+00  0.000000E+00       2527.32  0.000000E+00       2624.80       9944.39  0.000000E+00       12969.9       51.1090       10741.5 -1.000000E+10 -1.000000E+10 -1.000000E+10 -1.000000E+10 -1.000000E+10  0.000000E+00  0.000000E+00  0.000000E+00  0.000000E+00  0.000000E+00  0.000000E+00  0.000000E+00  0.000000E+00  0.000000E+00  0.000000E+00  0.000000E+00  0.000000E+00       Funtag_D
      29  4.884920E+05  4.420534E+05       46438.6       12.0387     0.0166796      0.190176      0.186539      0.240762    0.00273391   -0.00637060    0.00827611       1.01640       Opttag_D
      29       81.9562       1.17329       1.00000  0.000000E+00       1.00000  0.000000E+00      0.943216       91.8348       1.00000       38.8173  0.000000E+00       9.01894  0.000000E+00       9.01894  0.000000E+00  0.000000E+00      -18.0379       84.2583       3.97569        1       Scatag_D
      29       1930.39       5640.52  0.000000E+00  0.000000E+00       2527.93  0.000000E+00       2626.44       9938.32  0.000000E+00       12979.0       51.1911       10744.8 -1.000000E+10 -1.000000E+10 -1.000000E+10 -1.000000E+10 -1.000000E+10  0.000000E+00  0.000000E+00  0.000000E+00  0.000000E+00  0.000000E+00  0.000000E+00  0.000000E+00  0.000000E+00  0.000000E+00  0.000000E+00  0.000000E+00  0.000000E+00       Funtag_D
      30  4.884623E+05  4.420183E+05       46443.9       11.1526     0.0175695      0.201673      0.186525      0.240776    0.00282114   -0.00647642    0.00828014       1.01697       Opttag_D
      30       81.9596       1.17203       1.00000  0.000000E+00       1.00000  0.000000E+00      0.943186       91.8065       1.00000       38.8065  0.000000E+00       9.01837  0.000000E+00       9.01837  0.000000E+00  0.000000E+00      -18.0367       84.2583       3.97569        1       Scatag_D
      30       1932.27       5646.87  0.000000E+00  0.000000E+00       2528.49  0.000000E+00       2627.86       9923.02  0.000000E+00       12986.9       51.3187       10747.2 -1.000000E+10 -1.000000E+10 -1.000000E+10 -1.000000E+10 -1.000000E+10  0.000000E+00  0.000000E+00  0.000000E+00  0.000000E+00  0.000000E+00  0.000000E+00  0.000000E+00  0.000000E+00  0.000000E+00  0.000000E+00  0.000000E+00  0.000000E+00       Funtag_D
      31  4.884358E+05  4.419701E+05       46465.6       14.2211     0.0188618      0.218181      0.186504      0.240794    0.00294121   -0.00662351    0.00828549       1.01813       Opttag_D
      31       81.9657       1.17037       1.00000  0.000000E+00       1.00000  0.000000E+00      0.943144       91.7680       1.00000       38.7919  0.000000E+00       9.01771  0.000000E+00       9.01771  0.000000E+00  0.000000E+00      -18.0354       84.2583       3.97569        1       Scatag_D
      31       1934.76       5659.73  0.000000E+00  0.000000E+00       2529.25  0.000000E+00       2629.98       9910.76  0.000000E+00       12998.9       51.4911       10750.7 -1.000000E+10 -1.000000E+10 -1.000000E+10 -1.000000E+10 -1.000000E+10  0.000000E+00  0.000000E+00  0.000000E+00  0.000000E+00  0.000000E+00  0.000000E+00  0.000000E+00  0.000000E+00  0.000000E+00  0.000000E+00  0.000000E+00  0.000000E+00       Funtag_D
      32  4.884103E+05  4.419387E+05       46471.6       11.4010     0.0197151      0.229000      0.186488      0.240807    0.00301931   -0.00672303    0.00828656       1.01824       Opttag_D
      32       81.9708       1.16940       1.00000  0.000000E+00       1.00000  0.000000E+00      0.943120       91.7451       1.00000       38.7822  0.000000E+00       9.01728  0.000000E+00       9.01728  0.000000E+00  0.000000E+00      -18.0346       84.2583       3.97569        1       Scatag_D
      32       1935.26       5661.02  0.000000E+00  0.000000E+00       2529.87  0.000000E+00       2631.40       9902.31  0.000000E+00       13007.2       51.5866       10752.9 -1.000000E+10 -1.000000E+10 -1.000000E+10 -1.000000E+10 -1.000000E+10  0.000000E+00  0.000000E+00  0.000000E+00  0.000000E+00  0.000000E+00  0.000000E+00  0.000000E+00  0.000000E+00  0.000000E+00  0.000000E+00  0.000000E+00  0.000000E+00       Funtag_D
      33  4.883840E+05  4.419129E+05       46471.1       10.5488     0.0203772      0.237355      0.186475      0.240816    0.00308358   -0.00680299    0.00828743       1.01865       Opttag_D
      33       81.9762       1.16875       1.00000  0.000000E+00       1.00000  0.000000E+00      0.943103       91.7308       1.00000       38.7743  0.000000E+00       9.01702  0.000000E+00       9.01702  0.000000E+00  0.000000E+00      -18.0340       84.2583       3.97569        1       Scatag_D
      33       1935.67       5665.58  0.000000E+00  0.000000E+00       2530.31  0.000000E+00       2632.57       9886.84  0.000000E+00       13013.8       51.6286       10754.6 -1.000000E+10 -1.000000E+10 -1.000000E+10 -1.000000E+10 -1.000000E+10  0.000000E+00  0.000000E+00  0.000000E+00  0.000000E+00  0.000000E+00  0.000000E+00  0.000000E+00  0.000000E+00  0.000000E+00  0.000000E+00  0.000000E+00  0.000000E+00       Funtag_D
      34  4.883397E+05  4.418320E+05       46507.7       16.6964     0.0225358      0.264108      0.186433      0.240854    0.00328502   -0.00707209    0.00829853       1.02056       Opttag_D
      34       81.9947       1.16671       1.00000  0.000000E+00       1.00000  0.000000E+00      0.943050       91.6845       1.00000       38.7516  0.000000E+00       9.01610  0.000000E+00       9.01610  0.000000E+00  0.000000E+00      -18.0322       84.2583       3.97569        1       Scatag_D
      34       1940.86       5686.85  0.000000E+00  0.000000E+00       2531.79  0.000000E+00       2636.87       9862.07  0.000000E+00       13035.5       51.7629       10762.0 -1.000000E+10 -1.000000E+10 -1.000000E+10 -1.000000E+10 -1.000000E+10  0.000000E+00  0.000000E+00  0.000000E+00  0.000000E+00  0.000000E+00  0.000000E+00  0.000000E+00  0.000000E+00  0.000000E+00  0.000000E+00  0.000000E+00  0.000000E+00       Funtag_D
      35  4.883083E+05  4.417627E+05       46545.6       16.1556     0.0246016      0.289134      0.186397      0.240904    0.00345745   -0.00733546    0.00830402       1.02159       Opttag_D
      35       82.0129       1.16501       1.00000  0.000000E+00       1.00000  0.000000E+00      0.943002       91.6407       1.00000       38.7317  0.000000E+00       9.01522  0.000000E+00       9.01522  0.000000E+00  0.000000E+00      -18.0304       84.2583       3.97569        1       Scatag_D
      35       1943.43       5698.25  0.000000E+00  0.000000E+00       2533.08  0.000000E+00       2641.03       9853.64  0.000000E+00       13055.6       51.9347       10768.6 -1.000000E+10 -1.000000E+10 -1.000000E+10 -1.000000E+10 -1.000000E+10  0.000000E+00  0.000000E+00  0.000000E+00  0.000000E+00  0.000000E+00  0.000000E+00  0.000000E+00  0.000000E+00  0.000000E+00  0.000000E+00  0.000000E+00  0.000000E+00       Funtag_D
      36  4.882817E+05  4.417587E+05       46523.0       10.3082     0.0246560      0.289638      0.186395      0.240906    0.00346744   -0.00733906    0.00830235       1.02100       Opttag_D
      36       82.0143       1.16499       1.00000  0.000000E+00       1.00000  0.000000E+00      0.943001       91.6411       1.00000       38.7298  0.000000E+00       9.01517  0.000000E+00       9.01517  0.000000E+00  0.000000E+00      -18.0303       84.2583       3.97569        1       Scatag_D
      36       1942.65       5691.69  0.000000E+00  0.000000E+00       2533.14  0.000000E+00       2641.26       9837.74  0.000000E+00       13055.9       51.9322       10768.7 -1.000000E+10 -1.000000E+10 -1.000000E+10 -1.000000E+10 -1.000000E+10  0.000000E+00  0.000000E+00  0.000000E+00  0.000000E+00  0.000000E+00  0.000000E+00  0.000000E+00  0.000000E+00  0.000000E+00  0.000000E+00  0.000000E+00  0.000000E+00       Funtag_D
      37  4.882489E+05  4.417284E+05       46520.5       9.47996     0.0255034      0.299325      0.186378      0.240931    0.00354295   -0.00743161    0.00830755       1.02131       Opttag_D
      37       82.0233       1.16438       1.00000  0.000000E+00       1.00000  0.000000E+00      0.942984       91.6255       1.00000       38.7191  0.000000E+00       9.01481  0.000000E+00       9.01481  0.000000E+00  0.000000E+00      -18.0296       84.2583       3.97569        1       Scatag_D
      37       1945.08       5695.15  0.000000E+00  0.000000E+00       2533.74  0.000000E+00       2643.25       9815.83  0.000000E+00       13063.7       52.0000       10771.8 -1.000000E+10 -1.000000E+10 -1.000000E+10 -1.000000E+10 -1.000000E+10  0.000000E+00  0.000000E+00  0.000000E+00  0.000000E+00  0.000000E+00  0.000000E+00  0.000000E+00  0.000000E+00  0.000000E+00  0.000000E+00  0.000000E+00  0.000000E+00       Funtag_D
      38  4.882233E+05  4.416828E+05       46540.5       12.2885     0.0269501      0.315730      0.186349      0.240976    0.00365630   -0.00756622    0.00831396       1.02221       Opttag_D
      38       82.0379       1.16345       1.00000  0.000000E+00       1.00000  0.000000E+00      0.942953       91.5988       1.00000       38.7024  0.000000E+00       9.01416  0.000000E+00       9.01416  0.000000E+00  0.000000E+00      -18.0283       84.2583       3.97569        1       Scatag_D
      38       1948.08       5705.17  0.000000E+00  0.000000E+00       2534.80  0.000000E+00       2646.41       9800.02  0.000000E+00       13077.2       52.1201       10776.7 -1.000000E+10 -1.000000E+10 -1.000000E+10 -1.000000E+10 -1.000000E+10  0.000000E+00  0.000000E+00  0.000000E+00  0.000000E+00  0.000000E+00  0.000000E+00  0.000000E+00  0.000000E+00  0.000000E+00  0.000000E+00  0.000000E+00  0.000000E+00       Funtag_D
      39  4.881895E+05  4.416240E+05       46565.5       10.6159     0.0290080      0.338528      0.186313      0.241044    0.00380270   -0.00775189    0.00832075       1.02283       Opttag_D
      39       82.0585       1.16224       1.00000  0.000000E+00       1.00000  0.000000E+00      0.942909       91.5599       1.00000       38.6810  0.000000E+00       9.01341  0.000000E+00       9.01341  0.000000E+00  0.000000E+00      -18.0268       84.2583       3.97569        1       Scatag_D
      39       1951.27       5712.16  0.000000E+00  0.000000E+00       2536.28  0.000000E+00       2650.93       9781.84  0.000000E+00       13097.2       52.2919       10783.5 -1.000000E+10 -1.000000E+10 -1.000000E+10 -1.000000E+10 -1.000000E+10  0.000000E+00  0.000000E+00  0.000000E+00  0.000000E+00  0.000000E+00  0.000000E+00  0.000000E+00  0.000000E+00  0.000000E+00  0.000000E+00  0.000000E+00  0.000000E+00       Funtag_D
      40  4.881551E+05  4.415757E+05       46579.5       11.3867     0.0308349      0.357552      0.186282      0.241101    0.00392296   -0.00784376    0.00832480       1.02357       Opttag_D
      40       82.0776       1.16134       1.00000  0.000000E+00       1.00000  0.000000E+00      0.942875       91.5267       1.00000       38.6617  0.000000E+00       9.01273  0.000000E+00       9.01273  0.000000E+00  0.000000E+00      -18.0255       84.2583       3.97569        1       Scatag_D
      40       1953.17       5720.35  0.000000E+00  0.000000E+00       2537.65  0.000000E+00       2654.67       9757.31  0.000000E+00       13114.7       52.4624       10789.1 -1.000000E+10 -1.000000E+10 -1.000000E+10 -1.000000E+10 -1.000000E+10  0.000000E+00  0.000000E+00  0.000000E+00  0.000000E+00  0.000000E+00  0.000000E+00  0.000000E+00  0.000000E+00  0.000000E+00  0.000000E+00  0.000000E+00  0.000000E+00       Funtag_D
      41  4.881169E+05  4.415203E+05       46596.6       11.7366     0.0330781      0.379408      0.186246      0.241179    0.00406091   -0.00792675    0.00832883       1.02424       Opttag_D
      41       82.1017       1.16038       1.00000  0.000000E+00       1.00000  0.000000E+00      0.942838       91.4850       1.00000       38.6389  0.000000E+00       9.01206  0.000000E+00       9.01206  0.000000E+00  0.000000E+00      -18.0241       84.2583       3.97569        1       Scatag_D
      41       1955.06       5727.88  0.000000E+00  0.000000E+00       2539.44  0.000000E+00       2659.00       9731.20  0.000000E+00       13136.2       52.6727       10795.1 -1.000000E+10 -1.000000E+10 -1.000000E+10 -1.000000E+10 -1.000000E+10  0.000000E+00  0.000000E+00  0.000000E+00  0.000000E+00  0.000000E+00  0.000000E+00  0.000000E+00  0.000000E+00  0.000000E+00  0.000000E+00  0.000000E+00  0.000000E+00       Funtag_D
      42  4.880842E+05  4.414980E+05       46586.3       10.7655     0.0339080      0.385578      0.186232      0.241204    0.00411634   -0.00789594    0.00832905       1.02429       Opttag_D
      42       82.1118       1.16009       1.00000  0.000000E+00       1.00000  0.000000E+00      0.942832       91.4691       1.00000       38.6294  0.000000E+00       9.01166  0.000000E+00       9.01166  0.000000E+00  0.000000E+00      -18.0233       84.2583       3.97569        1       Scatag_D
      42       1955.16       5728.46  0.000000E+00  0.000000E+00       2540.00  0.000000E+00       2660.02       9709.80  0.000000E+00       13143.5       52.7561       10796.6 -1.000000E+10 -1.000000E+10 -1.000000E+10 -1.000000E+10 -1.000000E+10  0.000000E+00  0.000000E+00  0.000000E+00  0.000000E+00  0.000000E+00  0.000000E+00  0.000000E+00  0.000000E+00  0.000000E+00  0.000000E+00  0.000000E+00  0.000000E+00       Funtag_D
      43  4.880431E+05  4.414698E+05       46573.3       10.9614     0.0348806      0.390709      0.186211      0.241227    0.00418656   -0.00780317    0.00833089       1.02415       Opttag_D
      43       82.1247       1.15977       1.00000  0.000000E+00       1.00000  0.000000E+00      0.942836       91.4479       1.00000       38.6185  0.000000E+00       9.01102  0.000000E+00       9.01102  0.000000E+00  0.000000E+00      -18.0220       84.2583       3.97569        1       Scatag_D
      43       1956.03       5726.83  0.000000E+00  0.000000E+00       2540.73  0.000000E+00       2660.66       9687.04  0.000000E+00       13151.0       52.8461       10798.2 -1.000000E+10 -1.000000E+10 -1.000000E+10 -1.000000E+10 -1.000000E+10  0.000000E+00  0.000000E+00  0.000000E+00  0.000000E+00  0.000000E+00  0.000000E+00  0.000000E+00  0.000000E+00  0.000000E+00  0.000000E+00  0.000000E+00  0.000000E+00       Funtag_D
      44  4.880098E+05  4.414327E+05       46577.1       16.5928     0.0363767      0.398615      0.186183      0.241263    0.00427897   -0.00772613    0.00833479       1.02447       Opttag_D
      44       82.1442       1.15934       1.00000  0.000000E+00       1.00000  0.000000E+00      0.942836       91.4145       1.00000       38.6063  0.000000E+00       9.00966  0.000000E+00       9.00966  0.000000E+00  0.000000E+00      -18.0193       84.2583       3.97569        1       Scatag_D
      44       1957.86       5730.45  0.000000E+00  0.000000E+00       2541.78  0.000000E+00       2661.75       9669.03  0.000000E+00       13162.1       52.9272       10801.2 -1.000000E+10 -1.000000E+10 -1.000000E+10 -1.000000E+10 -1.000000E+10  0.000000E+00  0.000000E+00  0.000000E+00  0.000000E+00  0.000000E+00  0.000000E+00  0.000000E+00  0.000000E+00  0.000000E+00  0.000000E+00  0.000000E+00  0.000000E+00       Funtag_D
      45  4.879729E+05  4.414137E+05       46559.2       11.4276     0.0371506      0.403022      0.186170      0.241279    0.00432623   -0.00769184    0.00833785       1.02421       Opttag_D
      45       82.1545       1.15912       1.00000  0.000000E+00       1.00000  0.000000E+00      0.942838       91.3954       1.00000       38.6019  0.000000E+00       9.00871  0.000000E+00       9.00871  0.000000E+00  0.000000E+00      -18.0174       84.2583       3.97569        1       Scatag_D
      45       1959.30       5727.54  0.000000E+00  0.000000E+00       2542.22  0.000000E+00       2662.20       9644.65  0.000000E+00       13167.5       52.9317       10802.8 -1.000000E+10 -1.000000E+10 -1.000000E+10 -1.000000E+10 -1.000000E+10  0.000000E+00  0.000000E+00  0.000000E+00  0.000000E+00  0.000000E+00  0.000000E+00  0.000000E+00  0.000000E+00  0.000000E+00  0.000000E+00  0.000000E+00  0.000000E+00       Funtag_D
      46  4.879392E+05  4.413897E+05       46549.5       10.4448     0.0382399      0.419549      0.186152      0.241300    0.00438589   -0.00766968    0.00834240       1.02429       Opttag_D
      46       82.1685       1.15880       1.00000  0.000000E+00       1.00000  0.000000E+00      0.942833       91.3701       1.00000       38.5976  0.000000E+00       9.00730  0.000000E+00       9.00730  0.000000E+00  0.000000E+00      -18.0146       84.2583       3.97569        1       Scatag_D
      46       1961.43       5728.45  0.000000E+00  0.000000E+00       2542.87  0.000000E+00       2663.10       9620.53  0.000000E+00       13175.1       52.9127       10805.2 -1.000000E+10 -1.000000E+10 -1.000000E+10 -1.000000E+10 -1.000000E+10  0.000000E+00  0.000000E+00  0.000000E+00  0.000000E+00  0.000000E+00  0.000000E+00  0.000000E+00  0.000000E+00  0.000000E+00  0.000000E+00  0.000000E+00  0.000000E+00       Funtag_D
      47  4.879070E+05  4.413596E+05       46547.4       12.3099     0.0398327      0.443736      0.186129      0.241329    0.00446081   -0.00760907    0.00834769       1.02461       Opttag_D
      47       82.1895       1.15853       1.00000  0.000000E+00       1.00000  0.000000E+00      0.942823       91.3369       1.00000       38.5925  0.000000E+00       9.00497  0.000000E+00       9.00497  0.000000E+00  0.000000E+00      -18.0099       84.2583       3.97569        1       Scatag_D
      47       1963.92       5732.01  0.000000E+00  0.000000E+00       2543.78  0.000000E+00       2664.43       9596.42  0.000000E+00       13185.9       52.8978       10808.0 -1.000000E+10 -1.000000E+10 -1.000000E+10 -1.000000E+10 -1.000000E+10  0.000000E+00  0.000000E+00  0.000000E+00  0.000000E+00  0.000000E+00  0.000000E+00  0.000000E+00  0.000000E+00  0.000000E+00  0.000000E+00  0.000000E+00  0.000000E+00       Funtag_D
      48  4.878754E+05  4.413195E+05       46555.8       14.6709     0.0424513      0.483407      0.186097      0.241376    0.00456053   -0.00750077    0.00835458       1.02514       Opttag_D
      48       82.2245       1.15835       1.00000  0.000000E+00       1.00000  0.000000E+00      0.942808       91.2847       1.00000       38.5871  0.000000E+00       9.00107  0.000000E+00       9.00107  0.000000E+00  0.000000E+00      -18.0021       84.2583       3.97569        1       Scatag_D
      48       1967.16       5737.92  0.000000E+00  0.000000E+00       2545.15  0.000000E+00       2666.53       9570.11  0.000000E+00       13203.5       52.9094       10812.6 -1.000000E+10 -1.000000E+10 -1.000000E+10 -1.000000E+10 -1.000000E+10  0.000000E+00  0.000000E+00  0.000000E+00  0.000000E+00  0.000000E+00  0.000000E+00  0.000000E+00  0.000000E+00  0.000000E+00  0.000000E+00  0.000000E+00  0.000000E+00       Funtag_D
      49  4.878446E+05  4.413091E+05       46535.5       10.6291     0.0431784      0.494961      0.186089      0.241388    0.00458646   -0.00745467    0.00834977       1.02499       Opttag_D
      49       82.2352       1.15841       1.00000  0.000000E+00       1.00000  0.000000E+00      0.942802       91.2736       1.00000       38.5866  0.000000E+00       8.99956  0.000000E+00       8.99956  0.000000E+00  0.000000E+00      -17.9991       84.2583       3.97569        1       Scatag_D
      49       1964.90       5736.25  0.000000E+00  0.000000E+00       2545.59  0.000000E+00       2666.82       9549.06  0.000000E+00       13207.2       52.9303       10812.7 -1.000000E+10 -1.000000E+10 -1.000000E+10 -1.000000E+10 -1.000000E+10  0.000000E+00  0.000000E+00  0.000000E+00  0.000000E+00  0.000000E+00  0.000000E+00  0.000000E+00  0.000000E+00  0.000000E+00  0.000000E+00  0.000000E+00  0.000000E+00       Funtag_D
      50  4.878156E+05  4.412987E+05       46516.9       10.4550     0.0439692      0.508096      0.186078      0.241399    0.00461240   -0.00739484    0.00834554       1.02508       Opttag_D
      50       82.2475       1.15858       1.00000  0.000000E+00       1.00000  0.000000E+00      0.942798       91.2631       1.00000       38.5858  0.000000E+00       8.99769  0.000000E+00       8.99769  0.000000E+00  0.000000E+00      -17.9954       84.2583       3.97569        1       Scatag_D
      50       1962.91       5737.26  0.000000E+00  0.000000E+00       2546.00  0.000000E+00       2666.78       9528.21  0.000000E+00       13210.6       52.9768       10812.1 -1.000000E+10 -1.000000E+10 -1.000000E+10 -1.000000E+10 -1.000000E+10  0.000000E+00  0.000000E+00  0.000000E+00  0.000000E+00  0.000000E+00  0.000000E+00  0.000000E+00  0.000000E+00  0.000000E+00  0.000000E+00  0.000000E+00  0.000000E+00       Funtag_D
      51  4.877840E+05  4.412767E+05       46507.3       12.5167     0.0454957      0.532788      0.186058      0.241425    0.00466715   -0.00735582    0.00834238       1.02520       Opttag_D
      51       82.2702       1.15876       1.00000  0.000000E+00       1.00000  0.000000E+00      0.942789       91.2397       1.00000       38.5845  0.000000E+00       8.99442  0.000000E+00       8.99442  0.000000E+00  0.000000E+00      -17.9888       84.2583       3.97569        1       Scatag_D
      51       1961.42       5738.65  0.000000E+00  0.000000E+00       2546.73  0.000000E+00       2666.95       9509.85  0.000000E+00       13218.0       53.0543       10812.7 -1.000000E+10 -1.000000E+10 -1.000000E+10 -1.000000E+10 -1.000000E+10  0.000000E+00  0.000000E+00  0.000000E+00  0.000000E+00  0.000000E+00  0.000000E+00  0.000000E+00  0.000000E+00  0.000000E+00  0.000000E+00  0.000000E+00  0.000000E+00       Funtag_D
      52  4.877449E+05  4.412452E+05       46499.7       13.8898     0.0481309      0.574916      0.186025      0.241474    0.00474554   -0.00730038    0.00833837       1.02559       Opttag_D
      52       82.3093       1.15919       1.00000  0.000000E+00       1.00000  0.000000E+00      0.942772       91.2008       1.00000       38.5837  0.000000E+00       8.98904  0.000000E+00       8.98904  0.000000E+00  0.000000E+00      -17.9781       84.2583       3.97569        1       Scatag_D
      52       1959.54       5742.99  0.000000E+00  0.000000E+00       2548.09  0.000000E+00       2667.30       9483.74  0.000000E+00       13230.6       53.1798       10814.3 -1.000000E+10 -1.000000E+10 -1.000000E+10 -1.000000E+10 -1.000000E+10  0.000000E+00  0.000000E+00  0.000000E+00  0.000000E+00  0.000000E+00  0.000000E+00  0.000000E+00  0.000000E+00  0.000000E+00  0.000000E+00  0.000000E+00  0.000000E+00       Funtag_D
      53  4.877082E+05  4.412238E+05       46484.4       10.8895     0.0494996      0.596843      0.186005      0.241502    0.00479885   -0.00734277    0.00833955       1.02561       Opttag_D
      53       82.3292       1.15926       1.00000  0.000000E+00       1.00000  0.000000E+00      0.942764       91.1780       1.00000       38.5827  0.000000E+00       8.98632  0.000000E+00       8.98632  0.000000E+00  0.000000E+00      -17.9726       84.2583       3.97569        1       Scatag_D
      53       1960.09       5743.24  0.000000E+00  0.000000E+00       2548.75  0.000000E+00       2667.32       9459.21  0.000000E+00       13237.0       53.2053       10815.6 -1.000000E+10 -1.000000E+10 -1.000000E+10 -1.000000E+10 -1.000000E+10  0.000000E+00  0.000000E+00  0.000000E+00  0.000000E+00  0.000000E+00  0.000000E+00  0.000000E+00  0.000000E+00  0.000000E+00  0.000000E+00  0.000000E+00  0.000000E+00       Funtag_D
      54  4.876701E+05  4.411961E+05       46474.0       9.95020     0.0511285      0.623006      0.185982      0.241535    0.00486783   -0.00743161    0.00834162       1.02573       Opttag_D
      54       82.3528       1.15922       1.00000  0.000000E+00       1.00000  0.000000E+00      0.942753       91.1486       1.00000       38.5807  0.000000E+00       8.98335  0.000000E+00       8.98335  0.000000E+00  0.000000E+00      -17.9667       84.2583       3.97569        1       Scatag_D
      54       1961.07       5744.51  0.000000E+00  0.000000E+00       2549.53  0.000000E+00       2667.31       9436.34  0.000000E+00       13244.4       53.1875       10817.7 -1.000000E+10 -1.000000E+10 -1.000000E+10 -1.000000E+10 -1.000000E+10  0.000000E+00  0.000000E+00  0.000000E+00  0.000000E+00  0.000000E+00  0.000000E+00  0.000000E+00  0.000000E+00  0.000000E+00  0.000000E+00  0.000000E+00  0.000000E+00       Funtag_D
      55  4.876425E+05  4.411694E+05       46473.1       12.1470     0.0526847      0.648193      0.185959      0.241556    0.00493411   -0.00748514    0.00834234       1.02592       Opttag_D
      55       82.3753       1.15915       1.00000  0.000000E+00       1.00000  0.000000E+00      0.942744       91.1183       1.00000       38.5782  0.000000E+00       8.98057  0.000000E+00       8.98057  0.000000E+00  0.000000E+00      -17.9611       84.2583       3.97569        1       Scatag_D
      55       1961.41       5746.64  0.000000E+00  0.000000E+00       2550.39  0.000000E+00       2667.18       9424.34  0.000000E+00       13250.5       53.1262       10819.5 -1.000000E+10 -1.000000E+10 -1.000000E+10 -1.000000E+10 -1.000000E+10  0.000000E+00  0.000000E+00  0.000000E+00  0.000000E+00  0.000000E+00  0.000000E+00  0.000000E+00  0.000000E+00  0.000000E+00  0.000000E+00  0.000000E+00  0.000000E+00       Funtag_D
      56  4.876117E+05  4.411442E+05       46467.5       9.74082     0.0540913      0.671102      0.185939      0.241571    0.00499703   -0.00753755    0.00834357       1.02593       Opttag_D
      56       82.3954       1.15899       1.00000  0.000000E+00       1.00000  0.000000E+00      0.942736       91.0872       1.00000       38.5753  0.000000E+00       8.97792  0.000000E+00       8.97792  0.000000E+00  0.000000E+00      -17.9558       84.2583       3.97569        1       Scatag_D
      56       1961.98       5746.80  0.000000E+00  0.000000E+00       2551.15  0.000000E+00       2667.00       9410.55  0.000000E+00       13255.7       53.0437       10821.3 -1.000000E+10 -1.000000E+10 -1.000000E+10 -1.000000E+10 -1.000000E+10  0.000000E+00  0.000000E+00  0.000000E+00  0.000000E+00  0.000000E+00  0.000000E+00  0.000000E+00  0.000000E+00  0.000000E+00  0.000000E+00  0.000000E+00  0.000000E+00       Funtag_D
      57  4.875819E+05  4.411219E+05       46460.0       9.48520     0.0555564      0.695084      0.185920      0.241581    0.00505255   -0.00756555    0.00834108       1.02609       Opttag_D
      57       82.4163       1.15891       1.00000  0.000000E+00       1.00000  0.000000E+00      0.942732       91.0526       1.00000       38.5717  0.000000E+00       8.97495  0.000000E+00       8.97495  0.000000E+00  0.000000E+00      -17.9499       84.2583       3.97569        1       Scatag_D
      57       1960.81       5748.58  0.000000E+00  0.000000E+00       2551.97  0.000000E+00       2666.76       9396.12  0.000000E+00       13260.1       52.9677       10822.8 -1.000000E+10 -1.000000E+10 -1.000000E+10 -1.000000E+10 -1.000000E+10  0.000000E+00  0.000000E+00  0.000000E+00  0.000000E+00  0.000000E+00  0.000000E+00  0.000000E+00  0.000000E+00  0.000000E+00  0.000000E+00  0.000000E+00  0.000000E+00       Funtag_D
      58  4.875556E+05  4.411000E+05       46455.6       11.4610     0.0571685      0.721467      0.185898      0.241581    0.00510692   -0.00757072    0.00833747       1.02631       Opttag_D
      58       82.4392       1.15886       1.00000  0.000000E+00       1.00000  0.000000E+00      0.942730       91.0115       1.00000       38.5678  0.000000E+00       8.97133  0.000000E+00       8.97133  0.000000E+00  0.000000E+00      -17.9427       84.2583       3.97569        1       Scatag_D
      58       1959.12       5751.10  0.000000E+00  0.000000E+00       2552.97  0.000000E+00       2666.47       9385.68  0.000000E+00       13263.8       52.9422       10823.5 -1.000000E+10 -1.000000E+10 -1.000000E+10 -1.000000E+10 -1.000000E+10  0.000000E+00  0.000000E+00  0.000000E+00  0.000000E+00  0.000000E+00  0.000000E+00  0.000000E+00  0.000000E+00  0.000000E+00  0.000000E+00  0.000000E+00  0.000000E+00       Funtag_D
      59  4.875300E+05  4.410864E+05       46443.6       10.1693     0.0584534      0.742416      0.185884      0.241581    0.00514083   -0.00758219    0.00833535       1.02640       Opttag_D
      59       82.4571       1.15893       1.00000  0.000000E+00       1.00000  0.000000E+00      0.942730       90.9784       1.00000       38.5638  0.000000E+00       8.96827  0.000000E+00       8.96827  0.000000E+00  0.000000E+00      -17.9365       84.2583       3.97569        1       Scatag_D
      59       1958.12       5752.11  0.000000E+00  0.000000E+00       2553.76  0.000000E+00       2666.33       9369.92  0.000000E+00       13266.6       52.9629       10823.8 -1.000000E+10 -1.000000E+10 -1.000000E+10 -1.000000E+10 -1.000000E+10  0.000000E+00  0.000000E+00  0.000000E+00  0.000000E+00  0.000000E+00  0.000000E+00  0.000000E+00  0.000000E+00  0.000000E+00  0.000000E+00  0.000000E+00  0.000000E+00       Funtag_D
      60  4.875023E+05  4.410759E+05       46426.4       9.32278     0.0596421      0.761899      0.185871      0.241574    0.00516696   -0.00759006    0.00833049       1.02648       Opttag_D
      60       82.4736       1.15904       1.00000  0.000000E+00       1.00000  0.000000E+00      0.942733       90.9460       1.00000       38.5587  0.000000E+00       8.96526  0.000000E+00       8.96526  0.000000E+00  0.000000E+00      -17.9305       84.2583       3.97569        1       Scatag_D
      60       1955.84       5752.95  0.000000E+00  0.000000E+00       2554.54  0.000000E+00       2666.30       9352.05  0.000000E+00       13268.2       53.0202       10823.5 -1.000000E+10 -1.000000E+10 -1.000000E+10 -1.000000E+10 -1.000000E+10  0.000000E+00  0.000000E+00  0.000000E+00  0.000000E+00  0.000000E+00  0.000000E+00  0.000000E+00  0.000000E+00  0.000000E+00  0.000000E+00  0.000000E+00  0.000000E+00       Funtag_D
      61  4.874718E+05  4.410547E+05       46417.1       13.2769     0.0619492      0.799051      0.185844      0.241560    0.00521960   -0.00761739    0.00832880       1.02676       Opttag_D
      61       82.5048       1.15919       1.00000  0.000000E+00       1.00000  0.000000E+00      0.942738       90.8834       1.00000       38.5504  0.000000E+00       8.96010  0.000000E+00       8.96010  0.000000E+00  0.000000E+00      -17.9202       84.2583       3.97569        1       Scatag_D
      61       1955.04       5756.15  0.000000E+00  0.000000E+00       2556.07  0.000000E+00       2666.75       9333.21  0.000000E+00       13272.6       53.0497       10824.3 -1.000000E+10 -1.000000E+10 -1.000000E+10 -1.000000E+10 -1.000000E+10  0.000000E+00  0.000000E+00  0.000000E+00  0.000000E+00  0.000000E+00  0.000000E+00  0.000000E+00  0.000000E+00  0.000000E+00  0.000000E+00  0.000000E+00  0.000000E+00       Funtag_D
      62  4.874406E+05  4.410399E+05       46400.7       10.4030     0.0637946      0.828487      0.185823      0.241544    0.00525649   -0.00764528    0.00832978       1.02674       Opttag_D
      62       82.5292       1.15931       1.00000  0.000000E+00       1.00000  0.000000E+00      0.942742       90.8315       1.00000       38.5433  0.000000E+00       8.95592  0.000000E+00       8.95592  0.000000E+00  0.000000E+00      -17.9118       84.2583       3.97569        1       Scatag_D
      62       1955.50       5755.93  0.000000E+00  0.000000E+00       2557.32  0.000000E+00       2667.36       9310.00  0.000000E+00       13276.3       53.0554       10825.3 -1.000000E+10 -1.000000E+10 -1.000000E+10 -1.000000E+10 -1.000000E+10  0.000000E+00  0.000000E+00  0.000000E+00  0.000000E+00  0.000000E+00  0.000000E+00  0.000000E+00  0.000000E+00  0.000000E+00  0.000000E+00  0.000000E+00  0.000000E+00       Funtag_D
      63  4.874146E+05  4.410287E+05       46386.0       9.17112     0.0648495      0.845222      0.185812      0.241527    0.00528454   -0.00765349    0.00833082       1.02674       Opttag_D
      63       82.5426       1.15926       1.00000  0.000000E+00       1.00000  0.000000E+00      0.942746       90.7993       1.00000       38.5384  0.000000E+00       8.95388  0.000000E+00       8.95388  0.000000E+00  0.000000E+00      -17.9078       84.2583       3.97569        1       Scatag_D
      63       1955.99       5755.90  0.000000E+00  0.000000E+00       2558.05  0.000000E+00       2667.78       9290.64  0.000000E+00       13278.4       53.0112       10826.2 -1.000000E+10 -1.000000E+10 -1.000000E+10 -1.000000E+10 -1.000000E+10  0.000000E+00  0.000000E+00  0.000000E+00  0.000000E+00  0.000000E+00  0.000000E+00  0.000000E+00  0.000000E+00  0.000000E+00  0.000000E+00  0.000000E+00  0.000000E+00       Funtag_D
      64  4.873789E+05  4.410049E+05       46373.9       11.8357     0.0672436      0.882718      0.185788      0.241486    0.00534362   -0.00763797    0.00833758       1.02685       Opttag_D
      64       82.5722       1.15910       1.00000  0.000000E+00       1.00000  0.000000E+00      0.942755       90.7225       1.00000       38.5283  0.000000E+00       8.94929  0.000000E+00       8.94929  0.000000E+00  0.000000E+00      -17.8986       84.2583       3.97569        1       Scatag_D
      64       1959.17       5757.09  0.000000E+00  0.000000E+00       2559.76  0.000000E+00       2668.49       9264.44  0.000000E+00       13283.1       52.9562       10828.9 -1.000000E+10 -1.000000E+10 -1.000000E+10 -1.000000E+10 -1.000000E+10  0.000000E+00  0.000000E+00  0.000000E+00  0.000000E+00  0.000000E+00  0.000000E+00  0.000000E+00  0.000000E+00  0.000000E+00  0.000000E+00  0.000000E+00  0.000000E+00       Funtag_D
      65  4.873580E+05  4.409877E+05       46370.3       15.8298     0.0698804      0.923299      0.185767      0.241447    0.00538646   -0.00764123    0.00834648       1.02711       Opttag_D
      65       82.6041       1.15906       1.00000  0.000000E+00       1.00000  0.000000E+00      0.942765       90.6355       1.00000       38.5191  0.000000E+00       8.94410  0.000000E+00       8.94410  0.000000E+00  0.000000E+00      -17.8882       84.2583       3.97569        1       Scatag_D
      65       1963.35       5760.06  0.000000E+00  0.000000E+00       2561.70  0.000000E+00       2669.05       9241.85  0.000000E+00       13289.1       52.9818       10832.2 -1.000000E+10 -1.000000E+10 -1.000000E+10 -1.000000E+10 -1.000000E+10  0.000000E+00  0.000000E+00  0.000000E+00  0.000000E+00  0.000000E+00  0.000000E+00  0.000000E+00  0.000000E+00  0.000000E+00  0.000000E+00  0.000000E+00  0.000000E+00       Funtag_D
      66  4.873306E+05  4.409857E+05       46344.9       9.86193     0.0698950      0.923571      0.185765      0.241437    0.00539146   -0.00760761    0.00834385       1.02697       Opttag_D
      66       82.6036       1.15896       1.00000  0.000000E+00       1.00000  0.000000E+00      0.942767       90.6299       1.00000       38.5171  0.000000E+00       8.94414  0.000000E+00       8.94414  0.000000E+00  0.000000E+00      -17.8883       84.2583       3.97569        1       Scatag_D
      66       1962.11       5758.51  0.000000E+00  0.000000E+00       2561.77  0.000000E+00       2668.67       9220.55  0.000000E+00       13288.2       53.0366       10832.0 -1.000000E+10 -1.000000E+10 -1.000000E+10 -1.000000E+10 -1.000000E+10  0.000000E+00  0.000000E+00  0.000000E+00  0.000000E+00  0.000000E+00  0.000000E+00  0.000000E+00  0.000000E+00  0.000000E+00  0.000000E+00  0.000000E+00  0.000000E+00       Funtag_D
      67  4.872952E+05  4.409785E+05       46316.7       8.34637     0.0711276      0.942305      0.185756      0.241405    0.00540942   -0.00755138    0.00834407       1.02714       Opttag_D
      67       82.6172       1.15885       1.00000  0.000000E+00       1.00000  0.000000E+00      0.942775       90.5792       1.00000       38.5103  0.000000E+00       8.94179  0.000000E+00       8.94179  0.000000E+00  0.000000E+00      -17.8836       84.2583       3.97569        1       Scatag_D
      67       1962.22       5760.34  0.000000E+00  0.000000E+00       2562.82  0.000000E+00       2668.21       9187.16  0.000000E+00       13289.7       53.2117       10833.1 -1.000000E+10 -1.000000E+10 -1.000000E+10 -1.000000E+10 -1.000000E+10  0.000000E+00  0.000000E+00  0.000000E+00  0.000000E+00  0.000000E+00  0.000000E+00  0.000000E+00  0.000000E+00  0.000000E+00  0.000000E+00  0.000000E+00  0.000000E+00       Funtag_D
      68  4.872658E+05  4.409711E+05       46294.7       9.97072     0.0731060      0.971926      0.185744      0.241368    0.00542767   -0.00751618    0.00834682       1.02731       Opttag_D
      68       82.6395       1.15884       1.00000  0.000000E+00       1.00000  0.000000E+00      0.942785       90.5048       1.00000       38.5019  0.000000E+00       8.93817  0.000000E+00       8.93817  0.000000E+00  0.000000E+00      -17.8763       84.2583       3.97569        1       Scatag_D
      68       1963.51       5762.27  0.000000E+00  0.000000E+00       2564.47  0.000000E+00       2668.31       9153.80  0.000000E+00       13293.7       53.3903       10835.2 -1.000000E+10 -1.000000E+10 -1.000000E+10 -1.000000E+10 -1.000000E+10  0.000000E+00  0.000000E+00  0.000000E+00  0.000000E+00  0.000000E+00  0.000000E+00  0.000000E+00  0.000000E+00  0.000000E+00  0.000000E+00  0.000000E+00  0.000000E+00       Funtag_D
      69  4.872558E+05  4.409635E+05       46292.3       24.5832     0.0778690       1.04135      0.185721      0.241275    0.00544669   -0.00742340    0.00835788       1.02850       Opttag_D
      69       82.6922       1.15898       1.00000  0.000000E+00       1.00000  0.000000E+00      0.942812       90.3224       1.00000       38.4854  0.000000E+00       8.93037  0.000000E+00       8.93037  0.000000E+00  0.000000E+00      -17.8607       84.2583       3.97569        1       Scatag_D
      69       1968.72       5775.58  0.000000E+00  0.000000E+00       2568.60  0.000000E+00       2669.50       9109.93  0.000000E+00       13305.1       53.8355       10841.0 -1.000000E+10 -1.000000E+10 -1.000000E+10 -1.000000E+10 -1.000000E+10  0.000000E+00  0.000000E+00  0.000000E+00  0.000000E+00  0.000000E+00  0.000000E+00  0.000000E+00  0.000000E+00  0.000000E+00  0.000000E+00  0.000000E+00  0.000000E+00       Funtag_D
      70  4.872019E+05  4.409631E+05       46238.8       9.70859     0.0781868       1.04586      0.185721      0.241276    0.00544785   -0.00745298    0.00835510       1.02781       Opttag_D
      70       82.6959       1.15902       1.00000  0.000000E+00       1.00000  0.000000E+00      0.942812       90.3122       1.00000       38.4847  0.000000E+00       8.92988  0.000000E+00       8.92988  0.000000E+00  0.000000E+00      -17.8598       84.2583       3.97569        1       Scatag_D
      70       1967.41       5767.93  0.000000E+00  0.000000E+00       2568.88  0.000000E+00       2669.93       9063.32  0.000000E+00       13306.4       53.7880       10841.1 -1.000000E+10 -1.000000E+10 -1.000000E+10 -1.000000E+10 -1.000000E+10  0.000000E+00  0.000000E+00  0.000000E+00  0.000000E+00  0.000000E+00  0.000000E+00  0.000000E+00  0.000000E+00  0.000000E+00  0.000000E+00  0.000000E+00  0.000000E+00       Funtag_D
      71  4.871840E+05  4.409596E+05       46224.4       7.57747     0.0783400       1.04786      0.185720      0.241274    0.00545640   -0.00745545    0.00835480       1.02771       Opttag_D
      71       82.6975       1.15894       1.00000  0.000000E+00       1.00000  0.000000E+00      0.942814       90.3036       1.00000       38.4825  0.000000E+00       8.92993  0.000000E+00       8.92993  0.000000E+00  0.000000E+00      -17.8599       84.2583       3.97569        1       Scatag_D
      71       1967.27       5766.71  0.000000E+00  0.000000E+00       2569.08  0.000000E+00       2670.35       9049.42  0.000000E+00       13306.8       53.7417       10841.1 -1.000000E+10 -1.000000E+10 -1.000000E+10 -1.000000E+10 -1.000000E+10  0.000000E+00  0.000000E+00  0.000000E+00  0.000000E+00  0.000000E+00  0.000000E+00  0.000000E+00  0.000000E+00  0.000000E+00  0.000000E+00  0.000000E+00  0.000000E+00       Funtag_D
      72  4.871538E+05  4.409506E+05       46203.2       8.83138     0.0796101       1.06531      0.185708      0.241246    0.00547878   -0.00743038    0.00835717       1.02774       Opttag_D
      72       82.7114       1.15878       1.00000  0.000000E+00       1.00000  0.000000E+00      0.942824       90.2475       1.00000       38.4757  0.000000E+00       8.92874  0.000000E+00       8.92874  0.000000E+00  0.000000E+00      -17.8575       84.2583       3.97569        1       Scatag_D
      72       1968.39       5767.09  0.000000E+00  0.000000E+00       2570.38  0.000000E+00       2671.64       9019.92  0.000000E+00       13309.7       53.7059       10842.4 -1.000000E+10 -1.000000E+10 -1.000000E+10 -1.000000E+10 -1.000000E+10  0.000000E+00  0.000000E+00  0.000000E+00  0.000000E+00  0.000000E+00  0.000000E+00  0.000000E+00  0.000000E+00  0.000000E+00  0.000000E+00  0.000000E+00  0.000000E+00       Funtag_D
      73  4.871292E+05  4.409356E+05       46193.6       17.7704     0.0819284       1.09643      0.185690      0.241191    0.00551615   -0.00736965    0.00836271       1.02803       Opttag_D
      73       82.7372       1.15851       1.00000  0.000000E+00       1.00000  0.000000E+00      0.942844       90.1444       1.00000       38.4674  0.000000E+00       8.92634  0.000000E+00       8.92634  0.000000E+00  0.000000E+00      -17.8527       84.2583       3.97569        1       Scatag_D
      73       1971.00       5770.38  0.000000E+00  0.000000E+00       2572.79  0.000000E+00       2673.44       8992.40  0.000000E+00       13314.9       53.6908       10845.0 -1.000000E+10 -1.000000E+10 -1.000000E+10 -1.000000E+10 -1.000000E+10  0.000000E+00  0.000000E+00  0.000000E+00  0.000000E+00  0.000000E+00  0.000000E+00  0.000000E+00  0.000000E+00  0.000000E+00  0.000000E+00  0.000000E+00  0.000000E+00       Funtag_D
      74  4.870939E+05  4.409312E+05       46162.8       10.6549     0.0834125       1.11610      0.185680      0.241155    0.00552719   -0.00732152    0.00836488       1.02804       Opttag_D
      74       82.7539       1.15848       1.00000  0.000000E+00       1.00000  0.000000E+00      0.942854       90.0812       1.00000       38.4642  0.000000E+00       8.92481  0.000000E+00       8.92481  0.000000E+00  0.000000E+00      -17.8496       84.2583       3.97569        1       Scatag_D
      74       1972.02       5770.49  0.000000E+00  0.000000E+00       2574.29  0.000000E+00       2674.44       8952.32  0.000000E+00       13318.7       53.7245       10846.8 -1.000000E+10 -1.000000E+10 -1.000000E+10 -1.000000E+10 -1.000000E+10  0.000000E+00  0.000000E+00  0.000000E+00  0.000000E+00  0.000000E+00  0.000000E+00  0.000000E+00  0.000000E+00  0.000000E+00  0.000000E+00  0.000000E+00  0.000000E+00       Funtag_D
      75  4.870680E+05  4.409262E+05       46141.8       8.29147     0.0848783       1.13505      0.185672      0.241114    0.00553959   -0.00726124    0.00836712       1.02818       Opttag_D
      75       82.7710       1.15843       1.00000  0.000000E+00       1.00000  0.000000E+00      0.942866       90.0174       1.00000       38.4627  0.000000E+00       8.92324  0.000000E+00       8.92324  0.000000E+00  0.000000E+00      -17.8465       84.2583       3.97569        1       Scatag_D
      75       1973.07       5771.99  0.000000E+00  0.000000E+00       2575.78  0.000000E+00       2674.93       8921.92  0.000000E+00       13322.1       53.8052       10848.2 -1.000000E+10 -1.000000E+10 -1.000000E+10 -1.000000E+10 -1.000000E+10  0.000000E+00  0.000000E+00  0.000000E+00  0.000000E+00  0.000000E+00  0.000000E+00  0.000000E+00  0.000000E+00  0.000000E+00  0.000000E+00  0.000000E+00  0.000000E+00       Funtag_D
      76  4.870463E+05  4.409220E+05       46124.3       8.28062     0.0863837       1.15395      0.185661      0.241073    0.00555015   -0.00719490    0.00836924       1.02833       Opttag_D
      76       82.7893       1.15844       1.00000  0.000000E+00       1.00000  0.000000E+00      0.942877       89.9515       1.00000       38.4625  0.000000E+00       8.92153  0.000000E+00       8.92153  0.000000E+00  0.000000E+00      -17.8431       84.2583       3.97569        1       Scatag_D
      76       1974.08       5773.78  0.000000E+00  0.000000E+00       2577.37  0.000000E+00       2675.11       8895.19  0.000000E+00       13325.6       53.9260       10849.3 -1.000000E+10 -1.000000E+10 -1.000000E+10 -1.000000E+10 -1.000000E+10  0.000000E+00  0.000000E+00  0.000000E+00  0.000000E+00  0.000000E+00  0.000000E+00  0.000000E+00  0.000000E+00  0.000000E+00  0.000000E+00  0.000000E+00  0.000000E+00       Funtag_D
      77  4.870338E+05  4.409137E+05       46120.1       18.7085     0.0891926       1.18773      0.185643      0.240998    0.00557071   -0.00707569    0.00837276       1.02873       Opttag_D
      77       82.8257       1.15858       1.00000  0.000000E+00       1.00000  0.000000E+00      0.942902       89.8284       1.00000       38.4651  0.000000E+00       8.91822  0.000000E+00       8.91822  0.000000E+00  0.000000E+00      -17.8364       84.2583       3.97569        1       Scatag_D
      77       1975.74       5778.19  0.000000E+00  0.000000E+00       2580.38  0.000000E+00       2674.93       8874.09  0.000000E+00       13332.2       54.1896       10850.4 -1.000000E+10 -1.000000E+10 -1.000000E+10 -1.000000E+10 -1.000000E+10  0.000000E+00  0.000000E+00  0.000000E+00  0.000000E+00  0.000000E+00  0.000000E+00  0.000000E+00  0.000000E+00  0.000000E+00  0.000000E+00  0.000000E+00  0.000000E+00       Funtag_D
      78  4.870002E+05  4.409125E+05       46087.7       8.10074     0.0895686       1.19183      0.185641      0.240988    0.00557369   -0.00704882    0.00837099       1.02854       Opttag_D
      78       82.8315       1.15867       1.00000  0.000000E+00       1.00000  0.000000E+00      0.942905       89.8125       1.00000       38.4658  0.000000E+00       8.91758  0.000000E+00       8.91758  0.000000E+00  0.000000E+00      -17.8352       84.2583       3.97569        1       Scatag_D
      78       1974.90       5776.08  0.000000E+00  0.000000E+00       2580.82  0.000000E+00       2674.66       8844.11  0.000000E+00       13332.7       54.2328       10850.2 -1.000000E+10 -1.000000E+10 -1.000000E+10 -1.000000E+10 -1.000000E+10  0.000000E+00  0.000000E+00  0.000000E+00  0.000000E+00  0.000000E+00  0.000000E+00  0.000000E+00  0.000000E+00  0.000000E+00  0.000000E+00  0.000000E+00  0.000000E+00       Funtag_D
      79  4.869839E+05  4.409074E+05       46076.4       6.85435     0.0897140       1.19269      0.185635      0.240982    0.00558618   -0.00703049    0.00836867       1.02841       Opttag_D
      79       82.8356       1.15871       1.00000  0.000000E+00       1.00000  0.000000E+00      0.942908       89.8053       1.00000       38.4663  0.000000E+00       8.91712  0.000000E+00       8.91712  0.000000E+00  0.000000E+00      -17.8342       84.2583       3.97569        1       Scatag_D
      79       1973.81       5774.64  0.000000E+00  0.000000E+00       2581.05  0.000000E+00       2674.07       8837.41  0.000000E+00       13332.0       54.2561       10849.2 -1.000000E+10 -1.000000E+10 -1.000000E+10 -1.000000E+10 -1.000000E+10  0.000000E+00  0.000000E+00  0.000000E+00  0.000000E+00  0.000000E+00  0.000000E+00  0.000000E+00  0.000000E+00  0.000000E+00  0.000000E+00  0.000000E+00  0.000000E+00       Funtag_D
      80  4.869634E+05  4.408966E+05       46066.8       9.10089     0.0903243       1.19823      0.185624      0.240962    0.00561327   -0.00698483    0.00836608       1.02830       Opttag_D
      80       82.8477       1.15879       1.00000  0.000000E+00       1.00000  0.000000E+00      0.942919       89.7778       1.00000       38.4680  0.000000E+00       8.91587  0.000000E+00       8.91587  0.000000E+00  0.000000E+00      -17.8317       84.2583       3.97569        1       Scatag_D
      80       1972.58       5773.42  0.000000E+00  0.000000E+00       2581.83  0.000000E+00       2673.12       8832.19  0.000000E+00       13331.5       54.2939       10847.9 -1.000000E+10 -1.000000E+10 -1.000000E+10 -1.000000E+10 -1.000000E+10  0.000000E+00  0.000000E+00  0.000000E+00  0.000000E+00  0.000000E+00  0.000000E+00  0.000000E+00  0.000000E+00  0.000000E+00  0.000000E+00  0.000000E+00  0.000000E+00       Funtag_D
      81  4.869271E+05  4.408718E+05       46055.3       12.1070     0.0914845       1.20818      0.185601      0.240918    0.00567484   -0.00689947    0.00836312       1.02814       Opttag_D
      81       82.8717       1.15888       1.00000  0.000000E+00       1.00000  0.000000E+00      0.942942       89.7258       1.00000       38.4726  0.000000E+00       8.91340  0.000000E+00       8.91340  0.000000E+00  0.000000E+00      -17.8268       84.2583       3.97569        1       Scatag_D
      81       1971.19       5771.55  0.000000E+00  0.000000E+00       2583.28  0.000000E+00       2671.52       8827.41  0.000000E+00       13329.8       54.2698       10846.3 -1.000000E+10 -1.000000E+10 -1.000000E+10 -1.000000E+10 -1.000000E+10  0.000000E+00  0.000000E+00  0.000000E+00  0.000000E+00  0.000000E+00  0.000000E+00  0.000000E+00  0.000000E+00  0.000000E+00  0.000000E+00  0.000000E+00  0.000000E+00       Funtag_D
      82  4.869134E+05  4.408337E+05       46079.7       22.4974     0.0945885       1.23515      0.185559      0.240806    0.00576975   -0.00671539    0.00836089       1.02805       Opttag_D
      82       82.9318       1.15951       1.00000  0.000000E+00       1.00000  0.000000E+00      0.942994       89.5942       1.00000       38.4900  0.000000E+00       8.90709  0.000000E+00       8.90709  0.000000E+00  0.000000E+00      -17.8142       84.2583       3.97569        1       Scatag_D
      82       1970.14       5770.59  0.000000E+00  0.000000E+00       2586.94  0.000000E+00       2668.85       8853.48  0.000000E+00       13330.2       54.2706       10845.2 -1.000000E+10 -1.000000E+10 -1.000000E+10 -1.000000E+10 -1.000000E+10  0.000000E+00  0.000000E+00  0.000000E+00  0.000000E+00  0.000000E+00  0.000000E+00  0.000000E+00  0.000000E+00  0.000000E+00  0.000000E+00  0.000000E+00  0.000000E+00       Funtag_D
      83  4.868673E+05  4.408290E+05       46038.3       10.1217     0.0943875       1.23286      0.185557      0.240806    0.00578137   -0.00671010    0.00836033       1.02790       Opttag_D
      83       82.9295       1.15938       1.00000  0.000000E+00       1.00000  0.000000E+00      0.942994       89.6020       1.00000       38.4891  0.000000E+00       8.90734  0.000000E+00       8.90734  0.000000E+00  0.000000E+00      -17.8147       84.2583       3.97569        1       Scatag_D
      83       1969.87       5768.84  0.000000E+00  0.000000E+00       2586.72  0.000000E+00       2668.77       8815.70  0.000000E+00       13329.0       54.2328       10845.2 -1.000000E+10 -1.000000E+10 -1.000000E+10 -1.000000E+10 -1.000000E+10  0.000000E+00  0.000000E+00  0.000000E+00  0.000000E+00  0.000000E+00  0.000000E+00  0.000000E+00  0.000000E+00  0.000000E+00  0.000000E+00  0.000000E+00  0.000000E+00       Funtag_D
      84  4.868534E+05  4.408239E+05       46029.5       7.61248     0.0944665       1.23314      0.185552      0.240797    0.00579415   -0.00669537    0.00836026       1.02787       Opttag_D
      84       82.9322       1.15932       1.00000  0.000000E+00       1.00000  0.000000E+00      0.942997       89.5984       1.00000       38.4899  0.000000E+00       8.90702  0.000000E+00       8.90702  0.000000E+00  0.000000E+00      -17.8140       84.2583       3.97569        1       Scatag_D
      84       1969.84       5768.51  0.000000E+00  0.000000E+00       2586.80  0.000000E+00       2668.51       8808.13  0.000000E+00       13328.2       54.2193       10845.3 -1.000000E+10 -1.000000E+10 -1.000000E+10 -1.000000E+10 -1.000000E+10  0.000000E+00  0.000000E+00  0.000000E+00  0.000000E+00  0.000000E+00  0.000000E+00  0.000000E+00  0.000000E+00  0.000000E+00  0.000000E+00  0.000000E+00  0.000000E+00       Funtag_D
      85  4.868351E+05  4.408118E+05       46023.3       7.23913     0.0951020       1.25083      0.185540      0.240763    0.00582441   -0.00662812    0.00835966       1.02786       Opttag_D
      85       82.9469       1.15943       1.00000  0.000000E+00       1.00000  0.000000E+00      0.943009       89.5721       1.00000       38.4946  0.000000E+00       8.90528  0.000000E+00       8.90528  0.000000E+00  0.000000E+00      -17.8106       84.2583       3.97569        1       Scatag_D
      85       1969.56       5768.41  0.000000E+00  0.000000E+00       2587.52  0.000000E+00       2667.58       8803.38  0.000000E+00       13327.1       54.2311       10845.5 -1.000000E+10 -1.000000E+10 -1.000000E+10 -1.000000E+10 -1.000000E+10  0.000000E+00  0.000000E+00  0.000000E+00  0.000000E+00  0.000000E+00  0.000000E+00  0.000000E+00  0.000000E+00  0.000000E+00  0.000000E+00  0.000000E+00  0.000000E+00       Funtag_D
      86  4.868152E+05  4.407907E+05       46024.6       12.5742     0.0965150       1.29141      0.185517      0.240688    0.00587686   -0.00648294    0.00835738       1.02786       Opttag_D
      86       82.9796       1.15984       1.00000  0.000000E+00       1.00000  0.000000E+00      0.943034       89.5142       1.00000       38.5055  0.000000E+00       8.90121  0.000000E+00       8.90121  0.000000E+00  0.000000E+00      -17.8024       84.2583       3.97569        1       Scatag_D
      86       1968.48       5768.44  0.000000E+00  0.000000E+00       2589.17  0.000000E+00       2665.64       8806.92  0.000000E+00       13325.9       54.3166       10845.7 -1.000000E+10 -1.000000E+10 -1.000000E+10 -1.000000E+10 -1.000000E+10  0.000000E+00  0.000000E+00  0.000000E+00  0.000000E+00  0.000000E+00  0.000000E+00  0.000000E+00  0.000000E+00  0.000000E+00  0.000000E+00  0.000000E+00  0.000000E+00       Funtag_D
      87  4.867927E+05  4.407775E+05       46015.2       8.51407     0.0976440       1.32353      0.185502      0.240630    0.00590971   -0.00634260    0.00835572       1.02791       Opttag_D
      87       83.0058       1.16027       1.00000  0.000000E+00       1.00000  0.000000E+00      0.943055       89.4687       1.00000       38.5147  0.000000E+00       8.89793  0.000000E+00       8.89793  0.000000E+00  0.000000E+00      -17.7959       84.2583       3.97569        1       Scatag_D
      87       1967.70       5769.03  0.000000E+00  0.000000E+00       2590.46  0.000000E+00       2664.24       8797.94  0.000000E+00       13325.4       54.4027       10846.0 -1.000000E+10 -1.000000E+10 -1.000000E+10 -1.000000E+10 -1.000000E+10  0.000000E+00  0.000000E+00  0.000000E+00  0.000000E+00  0.000000E+00  0.000000E+00  0.000000E+00  0.000000E+00  0.000000E+00  0.000000E+00  0.000000E+00  0.000000E+00       Funtag_D
      88  4.867777E+05  4.407694E+05       46008.3       7.10956     0.0982989       1.34286      0.185493      0.240593    0.00592988   -0.00624723    0.00835409       1.02794       Opttag_D
      88       83.0217       1.16057       1.00000  0.000000E+00       1.00000  0.000000E+00      0.943067       89.4419       1.00000       38.5197  0.000000E+00       8.89579  0.000000E+00       8.89579  0.000000E+00  0.000000E+00      -17.7916       84.2583       3.97569        1       Scatag_D
      88       1966.93       5769.33  0.000000E+00  0.000000E+00       2591.22  0.000000E+00       2663.45       8791.78  0.000000E+00       13325.1       54.4704       10846.0 -1.000000E+10 -1.000000E+10 -1.000000E+10 -1.000000E+10 -1.000000E+10  0.000000E+00  0.000000E+00  0.000000E+00  0.000000E+00  0.000000E+00  0.000000E+00  0.000000E+00  0.000000E+00  0.000000E+00  0.000000E+00  0.000000E+00  0.000000E+00       Funtag_D
      89  4.867571E+05  4.407535E+05       46003.6       7.86493     0.0994342       1.37702      0.185478      0.240518    0.00596937   -0.00607102    0.00835249       1.02801       Opttag_D
      89       83.0500       1.16105       1.00000  0.000000E+00       1.00000  0.000000E+00      0.943090       89.3944       1.00000       38.5279  0.000000E+00       8.89195  0.000000E+00       8.89195  0.000000E+00  0.000000E+00      -17.7839       84.2583       3.97569        1       Scatag_D
      89       1966.18       5770.13  0.000000E+00  0.000000E+00       2592.55  0.000000E+00       2662.24       8787.49  0.000000E+00       13324.5       54.5707       10846.0 -1.000000E+10 -1.000000E+10 -1.000000E+10 -1.000000E+10 -1.000000E+10  0.000000E+00  0.000000E+00  0.000000E+00  0.000000E+00  0.000000E+00  0.000000E+00  0.000000E+00  0.000000E+00  0.000000E+00  0.000000E+00  0.000000E+00  0.000000E+00       Funtag_D
      90  4.867439E+05  4.407280E+05       46015.9       13.2891      0.101133       1.42771      0.185451      0.240402    0.00603277   -0.00580663    0.00835204       1.02814       Opttag_D
      90       83.0925       1.16175       1.00000  0.000000E+00       1.00000  0.000000E+00      0.943126       89.3240       1.00000       38.5409  0.000000E+00       8.88623  0.000000E+00       8.88623  0.000000E+00  0.000000E+00      -17.7725       84.2583       3.97569        1       Scatag_D
      90       1965.97       5771.55  0.000000E+00  0.000000E+00       2594.46  0.000000E+00       2660.85       8798.14  0.000000E+00       13324.0       54.6757       10846.2 -1.000000E+10 -1.000000E+10 -1.000000E+10 -1.000000E+10 -1.000000E+10  0.000000E+00  0.000000E+00  0.000000E+00  0.000000E+00  0.000000E+00  0.000000E+00  0.000000E+00  0.000000E+00  0.000000E+00  0.000000E+00  0.000000E+00  0.000000E+00       Funtag_D
      91  4.867236E+05  4.407220E+05       46001.6       7.76520      0.101279       1.43323      0.185448      0.240382    0.00604775   -0.00576716    0.00835246       1.02810       Opttag_D
      91       83.0972       1.16174       1.00000  0.000000E+00       1.00000  0.000000E+00      0.943132       89.3162       1.00000       38.5410  0.000000E+00       8.88554  0.000000E+00       8.88554  0.000000E+00  0.000000E+00      -17.7711       84.2583       3.97569        1       Scatag_D
      91       1966.17       5771.16  0.000000E+00  0.000000E+00       2594.63  0.000000E+00       2660.84       8784.62  0.000000E+00       13323.4       54.6683       10846.1 -1.000000E+10 -1.000000E+10 -1.000000E+10 -1.000000E+10 -1.000000E+10  0.000000E+00  0.000000E+00  0.000000E+00  0.000000E+00  0.000000E+00  0.000000E+00  0.000000E+00  0.000000E+00  0.000000E+00  0.000000E+00  0.000000E+00  0.000000E+00       Funtag_D
      92  4.867056E+05  4.407085E+05       45997.1       7.00569      0.101701       1.44766      0.185436      0.240338    0.00608147   -0.00567720    0.00835361       1.02805       Opttag_D
      92       83.1095       1.16177       1.00000  0.000000E+00       1.00000  0.000000E+00      0.943146       89.2964       1.00000       38.5424  0.000000E+00       8.88378  0.000000E+00       8.88378  0.000000E+00  0.000000E+00      -17.7676       84.2583       3.97569        1       Scatag_D
      92       1966.71       5770.63  0.000000E+00  0.000000E+00       2595.09  0.000000E+00       2660.76       8780.89  0.000000E+00       13322.5       54.6581       10845.9 -1.000000E+10 -1.000000E+10 -1.000000E+10 -1.000000E+10 -1.000000E+10  0.000000E+00  0.000000E+00  0.000000E+00  0.000000E+00  0.000000E+00  0.000000E+00  0.000000E+00  0.000000E+00  0.000000E+00  0.000000E+00  0.000000E+00  0.000000E+00       Funtag_D
      93  4.866903E+05  4.406926E+05       45997.7       7.90401      0.102335       1.46847      0.185422      0.240281    0.00612105   -0.00554664    0.00835457       1.02802       Opttag_D
      93       83.1274       1.16193       1.00000  0.000000E+00       1.00000  0.000000E+00      0.943164       89.2691       1.00000       38.5451  0.000000E+00       8.88119  0.000000E+00       8.88119  0.000000E+00  0.000000E+00      -17.7624       84.2583       3.97569        1       Scatag_D
      93       1967.16       5770.27  0.000000E+00  0.000000E+00       2595.76  0.000000E+00       2660.62       8782.08  0.000000E+00       13321.6       54.6814       10845.6 -1.000000E+10 -1.000000E+10 -1.000000E+10 -1.000000E+10 -1.000000E+10  0.000000E+00  0.000000E+00  0.000000E+00  0.000000E+00  0.000000E+00  0.000000E+00  0.000000E+00  0.000000E+00  0.000000E+00  0.000000E+00  0.000000E+00  0.000000E+00       Funtag_D

 An atom has moved more than     1.50 A from the initial/last
 position - if this was an accepted trial point, probably need to update the
 non-bonded contact list.

      94  4.866697E+05  4.406549E+05       46014.8       16.6248      0.104197       1.52825      0.185383      0.240120    0.00621480   -0.00519420    0.00835604       1.02795       Opttag_D
      94       83.1790       1.16262       1.00000  0.000000E+00       1.00000  0.000000E+00      0.943210       89.1947       1.00000       38.5522  0.000000E+00       8.87338  0.000000E+00       8.87338  0.000000E+00  0.000000E+00      -17.7468       84.2583       3.97569        1       Scatag_D
      94       1967.85       5769.47  0.000000E+00  0.000000E+00       2597.62  0.000000E+00       2660.34       8800.18  0.000000E+00       13320.4       54.8606       10844.0 -1.000000E+10 -1.000000E+10 -1.000000E+10 -1.000000E+10 -1.000000E+10  0.000000E+00  0.000000E+00  0.000000E+00  0.000000E+00  0.000000E+00  0.000000E+00  0.000000E+00  0.000000E+00  0.000000E+00  0.000000E+00  0.000000E+00  0.000000E+00       Funtag_D

 An atom has moved more than     1.50 A from the initial/last
 position - need to update the non-bonded contact list.


 ############################################################################################################
 ###################################################### setting up contact terms
 ############################################################################################################

      95  4.866389E+05  4.406391E+05       45999.8       9.32594      0.105233       1.56052      0.185364      0.240031    0.00625419   -0.00497986    0.00835664       1.02794       Opttag_D
      95       83.2070       1.16310       1.00000  0.000000E+00       1.00000  0.000000E+00      0.943234       89.1553       1.00000       38.5573  0.000000E+00       8.86919  0.000000E+00       8.86919  0.000000E+00  0.000000E+00      -17.7384       84.2583       3.97569        1       Scatag_D
      95       1968.14       5769.34  0.000000E+00  0.000000E+00       2598.59  0.000000E+00       2660.31       8784.98  0.000000E+00       13320.4       54.9923       10843.1 -1.000000E+10 -1.000000E+10 -1.000000E+10 -1.000000E+10 -1.000000E+10  0.000000E+00  0.000000E+00  0.000000E+00  0.000000E+00  0.000000E+00  0.000000E+00  0.000000E+00  0.000000E+00  0.000000E+00  0.000000E+00  0.000000E+00  0.000000E+00       Funtag_D
      96  4.866219E+05  4.406309E+05       45991.0       7.17020      0.105828       1.57918      0.185355      0.239980    0.00627455   -0.00486088    0.00835619       1.02792       Opttag_D
      96       83.2233       1.16342       1.00000  0.000000E+00       1.00000  0.000000E+00      0.943246       89.1339       1.00000       38.5585  0.000000E+00       8.86667  0.000000E+00       8.86667  0.000000E+00  0.000000E+00      -17.7333       84.2583       3.97569        1       Scatag_D
      96       1967.92       5769.08  0.000000E+00  0.000000E+00       2599.10  0.000000E+00       2660.15       8777.19  0.000000E+00       13320.6       55.1124       10841.9 -1.000000E+10 -1.000000E+10 -1.000000E+10 -1.000000E+10 -1.000000E+10  0.000000E+00  0.000000E+00  0.000000E+00  0.000000E+00  0.000000E+00  0.000000E+00  0.000000E+00  0.000000E+00  0.000000E+00  0.000000E+00  0.000000E+00  0.000000E+00       Funtag_D
      97  4.866049E+05  4.406186E+05       45986.3       7.31100      0.106698       1.60642      0.185341      0.239905    0.00630520   -0.00470626    0.00835647       1.02788       Opttag_D
      97       83.2469       1.16385       1.00000  0.000000E+00       1.00000  0.000000E+00      0.943265       89.1022       1.00000       38.5592  0.000000E+00       8.86306  0.000000E+00       8.86306  0.000000E+00  0.000000E+00      -17.7261       84.2583       3.97569        1       Scatag_D
      97       1968.05       5768.68  0.000000E+00  0.000000E+00       2599.82  0.000000E+00       2659.86       8773.69  0.000000E+00       13321.1       55.2889       10839.9 -1.000000E+10 -1.000000E+10 -1.000000E+10 -1.000000E+10 -1.000000E+10  0.000000E+00  0.000000E+00  0.000000E+00  0.000000E+00  0.000000E+00  0.000000E+00  0.000000E+00  0.000000E+00  0.000000E+00  0.000000E+00  0.000000E+00  0.000000E+00       Funtag_D
      98  4.865881E+05  4.405979E+05       45990.2       11.7011      0.108201       1.65273      0.185316      0.239780    0.00635659   -0.00447797    0.00835895       1.02784       Opttag_D
      98       83.2868       1.16452       1.00000  0.000000E+00       1.00000  0.000000E+00      0.943298       89.0467       1.00000       38.5595  0.000000E+00       8.85725  0.000000E+00       8.85725  0.000000E+00  0.000000E+00      -17.7145       84.2583       3.97569        1       Scatag_D
      98       1969.22       5768.24  0.000000E+00  0.000000E+00       2600.96  0.000000E+00       2659.22       8778.71  0.000000E+00       13322.8       55.5564       10835.5 -1.000000E+10 -1.000000E+10 -1.000000E+10 -1.000000E+10 -1.000000E+10  0.000000E+00  0.000000E+00  0.000000E+00  0.000000E+00  0.000000E+00  0.000000E+00  0.000000E+00  0.000000E+00  0.000000E+00  0.000000E+00  0.000000E+00  0.000000E+00       Funtag_D
      99  4.865660E+05  4.405847E+05       45981.3       7.95835      0.109102       1.68023      0.185300      0.239709    0.00638954   -0.00437474    0.00836117       1.02777       Opttag_D
      99       83.3104       1.16486       1.00000  0.000000E+00       1.00000  0.000000E+00      0.943318       89.0135       1.00000       38.5585  0.000000E+00       8.85380  0.000000E+00       8.85380  0.000000E+00  0.000000E+00      -17.7076       84.2583       3.97569        1       Scatag_D
      99       1970.27       5767.43  0.000000E+00  0.000000E+00       2601.59  0.000000E+00       2658.68       8771.33  0.000000E+00       13324.4       55.7157       10831.9 -1.000000E+10 -1.000000E+10 -1.000000E+10 -1.000000E+10 -1.000000E+10  0.000000E+00  0.000000E+00  0.000000E+00  0.000000E+00  0.000000E+00  0.000000E+00  0.000000E+00  0.000000E+00  0.000000E+00  0.000000E+00  0.000000E+00  0.000000E+00       Funtag_D

   MaxLik Scaling Cycle 0105

   Wilson plot

   Statistics in resolution bins after refinement cycle 0003
```


|  |  |
| --- | --- |
|  | - Rxpct factors plot- Rxpct factors table- <Log-Lik Gain>- Ln Sigma A- Correlation Coefficients |


```
   Iters     Tot_Funct    Xray_Funct    Geom_Funct          Grms     rmsD2init     maxD2init             R         Rfree           LLG       LLGfree     rms(bond)    rms(angle)
   Iters             K   B_IMPF_FRAG        K_MISS   B_TEMP_MISS   K_IMPF_MISS   B_IMPF_MISS        K_SOLV   B_TEMP_SOLV   K_IMPF_SOLV   B_IMPF_SOLV        B_TEMP     B_TEMP_11     B_TEMP_12     B_TEMP_22     B_TEMP_13     B_TEMP_23     B_TEMP_33      K_WILSON      B_WILSON      FGCYCLES
   Iters          BOND         ANGLE      IMPROPER       TORSION    SINTORSION      TRIGONAL        PLANAR  BCORRELATION       CONTACT     IDEALDIST        CHIRAL    SIMILARITY       OCCSSUM  UTILDISTANCE     UTILANGLE   UTILTORSION        RHOFIT        ADP-01        ADP-02        ADP-03        ADP-04        ADP-05        ADP-06        ADP-07        ADP-08        ADP-09        ADP-10      RHOFITPC            QM
     100  4.865459E+05  4.405713E+05       45974.6       7.71069      0.109842       1.70274      0.185285      0.239654    0.00642287   -0.00432650    0.00836375       1.02770       Opttag_D
     100       83.3296       1.16504       1.00000  0.000000E+00       1.00000  0.000000E+00      0.943336       88.9845       1.00000       38.5559  0.000000E+00       8.85107  0.000000E+00       8.85107  0.000000E+00  0.000000E+00      -17.7021       84.2583       3.97569        1       Scatag_D
     100       1971.49       5766.66  0.000000E+00  0.000000E+00       2602.04  0.000000E+00       2657.98       8766.98  0.000000E+00       13325.9       55.8277       10827.7 -1.000000E+10 -1.000000E+10 -1.000000E+10 -1.000000E+10 -1.000000E+10  0.000000E+00  0.000000E+00  0.000000E+00  0.000000E+00  0.000000E+00  0.000000E+00  0.000000E+00  0.000000E+00  0.000000E+00  0.000000E+00  0.000000E+00  0.000000E+00       Funtag_D
   Geometry Restraint Deviations SCREEN Information

 
 No -bcorrel_zeroocchydrogens_factor flag found - keeping default: will set B for OCC=0 hydrogens to    1.200 times heavy-atom B


 TLS information. Total number of groups:     2


 Group   Natoms    FixRB   FixTLS   Group Tag    Group Spec
     1     2781      Y        Y     tls1    { A|* }
     2     2770      Y        Y     tls2    { B|* }
 
NOTE BUSTER_TLS_SET tls1 { A|* }
NOTE BUSTER_TLS_T   tls1     -9.84122E-02  2.63375E-02  4.22310E-02 -5.87256E-02  1.84806E-02 -4.63797E-02
NOTE BUSTER_TLS_L   tls1      2.53184E+00  2.05865E+00  1.86779E+00 -8.79892E-01 -1.59062E+00  7.18718E-01
NOTE BUSTER_TLS_S   tls1     -1.17821E-02 -3.86196E-01  5.27491E-01 -4.35968E-01  2.67147E-01 -2.69276E-01  6.09961E-02 -3.35657E-01
NOTE BUSTER_TLS_O   tls1     -2.30832E+01 -8.41146E+01  1.12764E+01
NOTE BUSTER_TLS_FIX tls1 ALL
 
NOTE BUSTER_TLS_SET tls2 { B|* }
NOTE BUSTER_TLS_T   tls2     -1.73365E-01 -1.10956E-01  1.17182E-01  1.49139E-02  2.71700E-02 -2.71783E-02
NOTE BUSTER_TLS_L   tls2      1.57079E+00  1.30451E+00  1.13623E+00  8.10314E-01 -1.11889E+00 -8.20951E-01
NOTE BUSTER_TLS_S   tls2     -7.19371E-02  1.20184E-01 -9.00838E-02  1.24667E-02  1.65761E-01  7.66243E-02 -1.29102E-01  7.22209E-02
NOTE BUSTER_TLS_O   tls2      2.22817E+01 -7.23258E+01  3.51644E+00
NOTE BUSTER_TLS_FIX tls2 ALL

 For final position, Geometry function summary
     term                           number     weight       rms        GooF      function     fn/numb
     ================================================================================================
     BOND bond lengths (angs)       5665       2.000      0.0084       0.417      1971.49       0.348
     ANGL bond angles (degs)        7687       2.000        1.03       0.612      5766.66       0.750
     TORS split-harmonic tors       1275       0.000       18.70       1.176         0.00       0.000
     SINTOR sinusoidal tors         1275       2.000       18.70                  2602.04       2.041
     PLAN general plane (angs)       967       5.000      0.0148       0.741      2657.98       2.749
     BCORrelations (angs^2)         5665      10.000       1.102       0.393      8766.98       1.548
     CONT (bad contacts)               0       5.000                                 0.00
     IDEAL(ideal-dist contact)      5768       4.000                             13325.95
     CHIRAL (gelly semiharmon)       740       5.000                                55.83
     SIM similarity restraint for NCS and/or TARGET                              10827.66
     OCCSUM restraint on sum of occupanies                                           0.00       as none defined
     DISTAN utility restraints interatomic distance                                  0.00       as none defined
     UTILANGLE utility angle restraints                                              0.00       as none defined
     UTILTOR utility restraints on torsion angles                                    0.00       as none defined
     Peptide omega torsion angles                           2.98 degs - monitor only

 Weighted rms      0.0084         1.028  (temporary rms bond and angle for BUSTER to pick up!)
 Weighted intra-residual rms(bond) for StdProtein (excluding H)      0.0082
 Weighted intra-residual rms(bond) for Buffer     (excluding H)      0.0034
 Outputing geometry progress graph in plotmtv format to file:Geom_Res.mtv

 ############### Running final geometry/screen analysis (screen_final.txt)

 s/r gelly normal termination
```

Statistics and scale factors vs. cycle number
```

|  |  |  |
| --- | --- | --- |
|  | - Rxpct factors- <Log-Lik Gain>- GEOM Residuals- Ln Sigma_A_ | - Scale Factors- B Factors- Imperfection Factors |

```
   Final Geometry Information


---


### 2. Final results

Statistics and scale factors vs. cycle number
```

|  |  |  |
| --- | --- | --- |
|  | - Rxpct factors- <Log-Lik Gain>- GEOM Residuals- Ln Sigma_A_ | - Scale Factors- B Factors- Imperfection Factors |

```
   Average Figure of Merit
   Structure Factor Amplitudes


 # normal termination

  Program stopped.
```

closesok message: time spent in tree = 1739.42 (user) 32.15 (sys)

---

# Normal termination

```
 Resources usage summary:
     Time spent in client ...................  1739.43 (user)       32.17 (system)
     Time spent in scripts (    27 calls) ...     2.15 (user)        0.15 (system)
     Time spent in server ...................     0.00 (user)        0.00 (system)

     Max resident memory used (kb) .......... 383508
     Number of page faults .................. 0 (major)    1040461 (minor)
     Number of context switches ............. 13667 (niced)    6805 (other)
```
